# Clinical course of untreated cervical intraepithelial neoplasia grade 2 under active surveillance: systematic review and meta-analysis

**DOI:** 10.1136/bmj.k499

**Published:** 2018-02-27

**Authors:** Karoliina Tainio, Antonios Athanasiou, Kari A O Tikkinen, Riikka Aaltonen, Jovita Cárdenas, Hernándes, Sivan Glazer-Livson, Maija Jakobsson, Kirsi Joronen, Mari Kiviharju, Karolina Louvanto, Sanna Oksjoki, Riikka Tähtinen, Seppo Virtanen, Pekka Nieminen, Maria Kyrgiou, Ilkka Kalliala

**Affiliations:** 1 Department of Obstetrics and Gynaecology, University of Helsinki and Helsinki University Hospital, Helsinki, Finland; 2 Department of Obstetrics and Gynaecology, University Hospital of Ioannina, Ioannina, Greece; 3 Departments of Urology and Public Health, University of Helsinki and Helsinki University Hospital, Helsinki, Finland; 4 Department of Obstetrics and Gynaecology, Turku University Hospital and University of Turku, Turku, Finland; 5 National Center for Health Technology Excellence (CENETEC) Direction of Health Technologies assessment, Mexico City, Mexico; 6 Wolfson Institute of Preventive Medicine, Queen Mary University of London, London, UK; 7 Department of Obstetrics and Gynaecology, Kuopio University Hospital, Kuopio, Finland; 8 Institute of Reproduction and Developmental Biology, Department of Surgery & Cancer, Imperial College, London W12 0NN, UK; 9 West London Gynaecological Cancer Center, Queen Charlotte’s & Chelsea–Hammersmith Hospital, Imperial Healthcare NHS Trust, London, UK

## Abstract

**Objective:**

To estimate the regression, persistence, and progression of untreated cervical intraepithelial neoplasia grade 2 (CIN2) lesions managed conservatively as well as compliance with follow-up protocols.

**Design:**

Systematic review and meta-analysis.

**Data sources:**

Medline, Embase, and the Cumulative Index to Nursing and Allied Health Literature (CINAHL) from 1 January 1973 to 20 August 2016.

**Eligibility criteria:**

Studies reporting on outcomes of histologically confirmed CIN2 in non-pregnant women, managed conservatively for three or more months.

**Data synthesis:**

Two reviewers extracted data and assessed risk of bias. Random effects model was used to calculate pooled proportions for each outcome, and heterogeneity was assessed using I^2^ statistics.

**Main outcome measures:**

Rates of regression, persistence, or progression of CIN2 and default rates at different follow-up time points (3, 6, 12, 24, 36, and 60 months).

**Results:**

36 studies that included 3160 women were identified (seven randomised trials, 16 prospective cohorts, and 13 retrospective cohorts; 50% of the studies were at low risk of bias). At 24 months, the pooled rates were 50% (11 studies, 819/1470 women, 95% confidence interval 43% to 57%; I^2^=77%) for regression, 32% (eight studies, 334/1257 women, 23% to 42%; I^2^=82%) for persistence, and 18% (nine studies, 282/1445 women, 11% to 27%; I^2^=90%) for progression. In a subgroup analysis including 1069 women aged less than 30 years, the rates were 60% (four studies, 638/1069 women, 57% to 63%; I^2^=0%), 23% (two studies, 226/938 women, 20% to 26%; I^2^=97%), and 11% (three studies, 163/1033 women, 5% to 19%; I^2^=67%), respectively. The rate of non-compliance (at six to 24 months of follow-up) in prospective studies was around 10%.

**Conclusions:**

Most CIN2 lesions, particularly in young women (<30 years), regress spontaneously. Active surveillance, rather than immediate intervention, is therefore justified, especially among young women who are likely to adhere to monitoring.

**Systematic review registration:**

PROSPERO 2014: CRD42014014406.

## Introduction

Organised cervical cancer screening has led to a noticeable reduction in the incidence of and mortality from invasive cervical cancer, as pre-invasive lesions (cervical intraepithelial neoplasia, CIN) can be detected and treated appropriately.^1^ ^2^ Low grade squamous intraepithelial lesion (LSIL, also known as CIN1) is now recognised as a histological diagnosis of benign viral replication that should be managed conservatively, whereas CIN3 is recognised as a true pre-invasive precursor with a potential to progress to cancer. The clinical course and biological behaviour of CIN2 is less well understood.

Histological diagnosis of CIN2 or worse on a biopsy sample has been considered the cut-off point to proceed to treatment. Approximately 1.5 per 1000 women in developed countries are diagnosed as having CIN2/3 annually and the incidence is highest among women aged between 25 and 29 years—that is, 8.1 per 1000 women.^3^ Awareness that CIN2 is an equivocal histological diagnosis is increasing, and some studies have documented high spontaneous regression rates, particularly in young women.^4^ ^5^ In a prospective cohort study among 95 women aged 18 to 23, the regression rate was 63%, while only 15% of women progressed to CIN3 within three years.^6^ In another prospective cohort of 5052 women aged 18 to 62, 40% of CIN2 lesions regressed within two years,^7^ whereas the regression rate of CIN3 has been estimated to be around 32% and the progression to invasive cancer as high as 12%.^8^ Despite evidence on differences in the clinical course of CIN2 and CIN3, the updated World Health Organization 2014 histopathological classification graded these lesions as a single entity: high grade squamous intraepithelial lesion (HSIL).^9^

CIN2 and CIN3 are often treated with local excision of the cervix, which has proved to be effective.^10^ However, cervical treatment increases the risk of preterm birth and mid-trimester loss for women who go on to conceive after treatment.^11^ ^12^ ^13^ ^14^ ^15^ ^16^ As women undergoing local treatment for CIN are often of similar age as women having their first child, it is important to avoid overtreatment.

The high regression rates of CIN2 in some studies together with the morbidity associated with treatment has led to the adoption of alternative conservative management strategies in adolescent and young women. To date, however, no systematic reviews have explored the clinical course of histologically confirmed CIN2 lesions monitored conservatively. We performed a systematic review and meta-analysis on regression, progression, and persistence rates and adherence to follow-up in women with histologically confirmed CIN2 lesions managed with active surveillance.

## Methods

### Eligibility criteria and outcome measures

We included original studies that reported on outcomes of women with histologically proved CIN2 who were not treated at diagnosis, were monitored for three or more months, and had a diagnosis available at the end of the study period. We preferred histology to cytology for the diagnosis of the disease grade during the follow-up period; if histology was not available—particularly in the case of normal findings—we accepted the cytological diagnosis. We excluded studies on pregnant or women positive for antibodies to HIV, studies including fewer than 10 patients to complete surveillance, studies not defining the length of the follow-up period or merging CIN2 with another histological diagnosis (CIN1 or CIN3), and studies not published in English.

We explored disease outcomes for different time points that included regression (CIN1 or less), persistence (CIN2), and progression rates (CIN3 or worse). Furthermore, we explored the rate of non-compliance with active surveillance. The studies were broadly grouped and analysed based on the length of surveillance (3, 6, 12, 24, 36, and 60 months). These time points were based on the exact follow-up or the median or mean follow-up time. The outcome data were then included in the follow-up time point closest to the reported mean or median value.

### Literature search and data extraction

We searched three databases (Medline, Embase, and the Cumulative Index to Nursing and Allied Health Literature (CINAHL)) for publications between 1 January 1973 (when CIN grading was introduced)^17^ and 20 August 2016 (see supplementary file for details of the search strategy). We also hand searched the reference lists of all included studies.

From each study we extracted data on progression, persistence, and regression rates as well as the first author, year of publication, the design and setting, geographical region, the total number of participants, the number of participants with the outcomes of interest at different time points, and the number of participants with high risk human papillomavirus (HrHPV) or HPV16/18, or both at the beginning of follow-up, if available.

We accepted the definition of progression, persistence, and regression used in each study, recognising that there would be heterogeneity in definitions across studies. The regression and persistence definitions were classified into two broad groups: strict or lenient. We defined strict regression criteria as cytological and/or histological regression to normal, and lenient criteria as any regressive disease to cytological and/or histological diagnosis to atypical squamous cells of unknown significance (ASC-US) and to low grade squamous cervical intraepithelial lesion (LSIL). The strict persistence criterion included cytological or histological persistence of ASC-US, LSIL, or CIN2, whereas for the lenient criterion we considered only histological CIN2 and/or cytological high grade squamous cervical intraepithelial lesion (HSIL) and atypical squamous cells, cannot exclude HSIL (ASC-H).

### Risk of bias assessment

We assessed the risk of bias using a modified version of the Cochrane Collaboration’s risk of bias tool (see supplementary table 1). We evaluated each study according to five criteria: representativeness of population, assessment of exposure, presence of the outcome at the start of study, assessment of the outcome, and loss to follow-up. For each criterion we judged studies to have either a high risk or a low risk of bias. We classified studies at high risk of bias overall if at least one criterion was at high risk of bias. We defined loss to follow-up as the number of women lost to follow-up in prospective studies and as the number of initially eligible participants with missing data in retrospective cohort studies.

Two investigators independently performed literature searches, data extraction, and risk of bias assessment in duplicate. Disagreements were resolved by discussion and, if required, consensus was reached with the involvement of a third investigator. We registered the protocol (PROSPERO 2014: CRD42014014406) and followed the preferred reporting items for systematic reviews and meta-analysis guidance (PRISMA).^18^

### Data synthesis and assessment of heterogeneity

We defined regression, persistence, progression, and default rates as the ratio of observed number of women with a given outcome divided by the number of women attending in that follow-up time point. In case a single study presented more than one definition for an outcome, we used the most stringent definition given in the main analyses. Using the *metaprop* command in STATA^19^ we meta-analysed pooled proportions separately at the 3, 6, 12, 24, 36, and 60 month follow-up for each outcome. We used the exact binomial score test-based confidence intervals with the Freeman-Tukey double arcsine method to stabilise the variances for individual studies, in which many of the proportions were close to or at the margins of the possible interval (0 or 100%).^19^

The heterogeneity between studies was assessed with the I^2^ metric of inconsistency.^20^ If at least 10 studies were included in the meta-analysis, we used visual inspection of funnel plots and the Egger’s regression asymmetry test (P<0.10)^21^ to examine the possible presence of small study effects.

To explore the possible differences in summary estimates, we performed a single predefined sensitivity analysis using the lenient criteria (predefined hypothesis of higher regression and lower persistence rates than when using the strict criteria). To explore the possible sources of heterogeneity, we performed four predefined sensitivity analyses all with predefined hypothesis of reduced heterogeneity: including only the same uniform outcome definition criteria across all studies, including only studies with strictly defined follow-up time points, including only prospective studies, and including only low risk of bias studies. To further explore the sources of heterogeneity and the possible differences in summary estimates, we additionally performed subgroup analyses according to continent and the decade when the study was performed (to explore heterogeneity by differing diagnostics in different periods and different geographical locations), according to the age range (only ≤30 years, and studies with only ≤30 years excluded, respectively) and median age (≤30 years and >30 years), expecting to see more frequent regression and less frequent progression in younger patients, and according to the baseline HrHPV (positive or negative) or HPV16/18 (positive or negative) status of the women, expecting to see less frequent regression and more frequent progression in women positive for HrHPV or HPV16/18.

All analyses were performed in STATA version 13 (StataCorp, College Station, TX).

### Patient involvement

No patients were involved in the design, development of outcome measures, or conduct of the study. The results will be disseminated to the lay audience through the authors’ involvement with charities and through public presentations.

## Results

**Fig 1:**
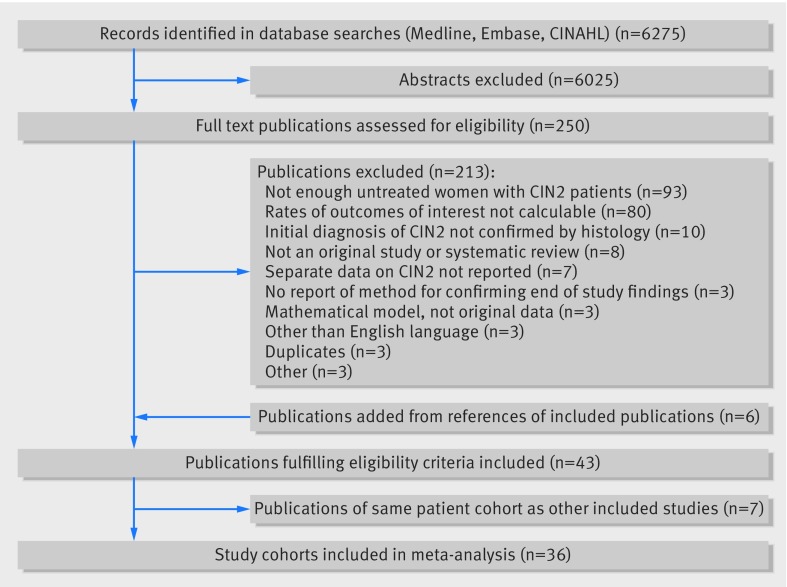
Flowchart outlining literature search and publication evaluation process

We identified 250 potentially eligible studies; 43 publications met the inclusion criteria (fig 1),^6^ ^22^ ^23^ ^24^ ^25^ ^26^ ^27^ ^28^ ^29^ ^30^ ^31^ ^32^ ^33^ ^34^ ^35^ ^36^ ^37^ ^38^ ^39^ ^40^ ^41^ ^42^ ^43^ ^44^ ^45^ ^46^ ^47^ ^48^ ^49^ ^50^ ^51^ ^52^ ^53^ ^54^ ^55^ ^56^ ^57^ ^58^ ^59^ ^60^ ^61^ ^62^ ^63^ but seven were duplicate reports of the same data.^27^ ^28^ ^31^ ^40^ ^50^ ^52^ ^54^ Of the 36 eligible studies (total of 3160 women), seven (19%) were randomised trials with suitable data in the non-experimental arm,^22^ ^25^ ^33^ ^39^ ^45^ ^57^ ^62^ 16 (44%) were prospective cohort studies,^6^ ^26^ ^30^ ^32^ ^34^ ^35^ ^36^ ^37^ ^38^ ^41^ ^43^ ^51^ ^55^ ^56^ ^60^ ^63^ and 13 (36%) were retrospective cohort studies^23^ ^24^ ^29^ ^42^ ^44^ ^46^ ^47^ ^48^ ^49^ ^53^ ^58^ ^59^ ^61^ (see supplementary table 2). The median follow-up was 16 months (range 3-72, interquartile range 7.6-27.4 months). The largest study included 924 women and the smallest included 12 women. Most studies (n=29, 81%) were small, with fewer than 100 women, and seven (19%) studies included only women aged less than 25.^6^ ^29^ ^42^ ^44^ ^48^ ^49^ ^53^ Twenty nine (81%) studies defined progression as a histological diagnosis of CIN3 or worse, whereas in seven of the 36 (19%) studies, worsening cytology was considered sufficient. Twenty five studies (69%) defined regression as normal histology or cytology, or both, whereas 17 (47%) considered that biopsy confirmed CIN1 or cytology suggestive of ASC-US or LSIL represented a regressive CIN2 lesion. Six (16%) studies reported results using both definitions (“complete” and “partial regression”) or provided the individual data on all cytology and histology outcomes. The number of studies reporting outcomes at different time points varied: 7 (19%) provided data at six months, 17 (47%) at 12 months, 14 (39%) at 24 months, 7 (19%) at 36 months, and 6 (17%) at 60 months. Data stratified according to the presence of HPV at baseline were available in 11 (31%) studies, but the follow-up time points and methods to define the presence of HPV varied between studies.

Eighteen of the 36 (50%) studies met the criteria for high risk of bias (see supplementary table 3). The most common causes for high risk of bias were loss to follow-up (n=14, 78%) and assessment of the outcome (n=5, 28%). A follow-up protocol was reported in 32 studies (see supplementary table 2). Colposcopy was routinely performed at every follow-up visit in at least 20 out of 36 studies. Biopsy samples were taken routinely at least once during the follow-up in five studies, and for histological confirmation of lesions even if they were not suspected to be CIN3 or worse in five studies. The protocols varied greatly between the studies, and of the 32 studies reporting the protocol, the definite criteria for colposcopic evaluation or histological sampling during the follow-up could not be defined in 11 studies. In the prospective, low risk of bias studies,^22^ ^25^ ^26^ ^30^ ^31^ ^33^ ^35^ ^36^ ^37^ ^38^ ^43^ ^47^ ^51^ ^56^ ^57^ ^63^ the most typical protocols included cytology and colposcopy every three to four months, with routine biopsies or biopsies when progression was suspected.

### Progression, persistence, and regression rates

**Fig 2:**
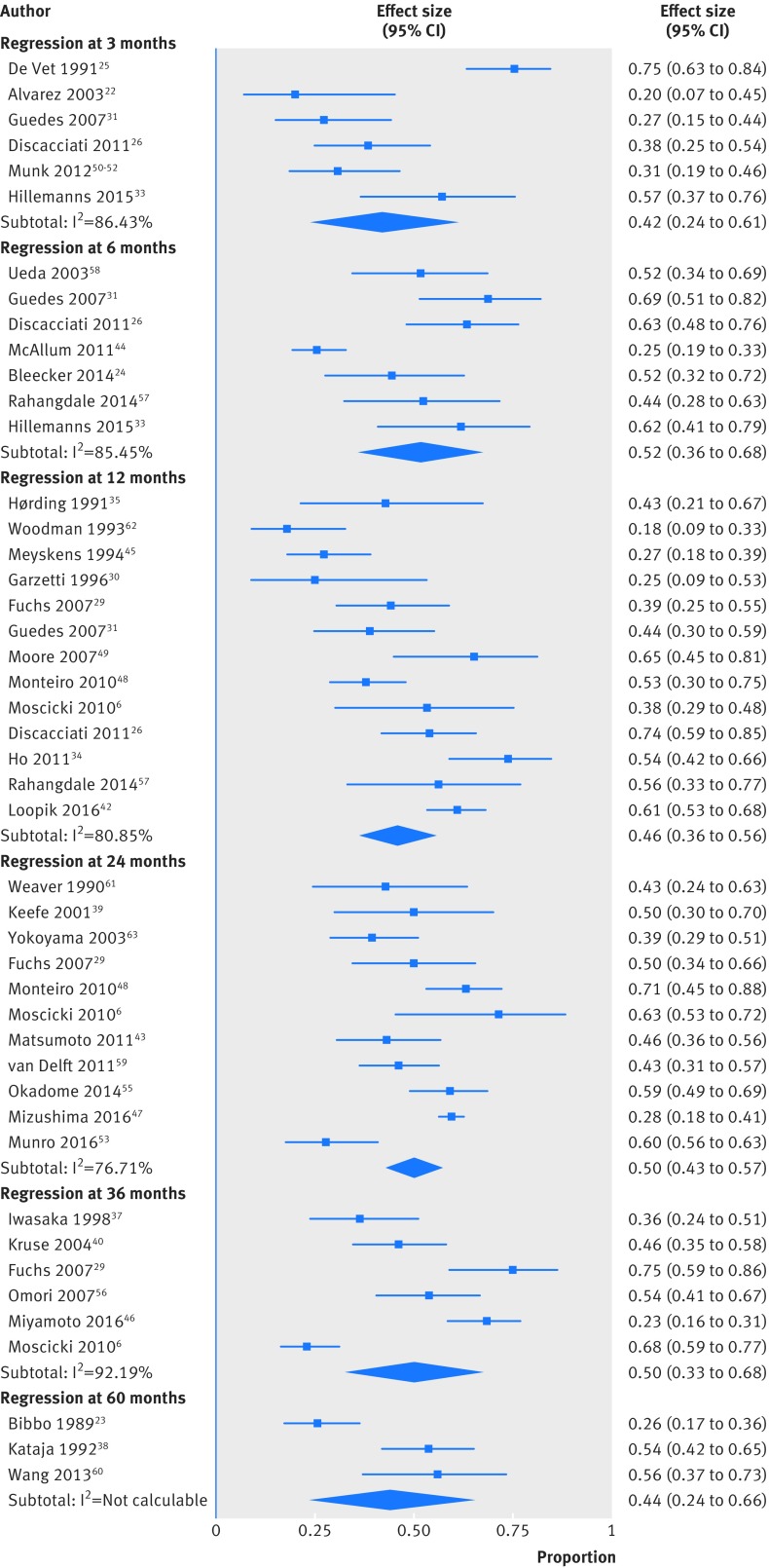
Regression rates of untreated cervical intraepithelial neoplasia grade 2 (CIN2) at different follow-up time points

The regression rate for histologically confirmed CIN2 lesions was high at all time points (table 1, fig 2, and supplementary table 4). The rate at 12 months was 46% (13 studies, 300/628 women, 95% confidence interval 36% to 56%; I^2^=81%) and at 24 months was 50% (11 studies, 819/1470 women, 43% to 57%; I^2^=77%). The number of studies for the remaining time points was smaller. At the extremes of surveillance (three months and 60 months), the regression rates were 42% (six studies, 97/208 women, 24% to 61%; I^2^=86%) and 44% (three studies, 70/170 women, 24% to 66%; I^2^=86%), respectively. Interstudy heterogeneity was substantial at all follow-up time points.

**Table 1:** Pooled rates of regression, persistence, and progression of CIN2 treated with active surveillance

Analysis	6 months				12 months				24 months		
	Regression	Persistence	Progression		Regression	Persistence	Progression		Regression	Persistence	Progression
Main analysis*:											
No of studies; n/N†	7; 139/328	5; 96/278	5; 42/278		13; 300/628	9; 110/414	13; 131/834		11; 819/1470	8; 334/1257	9; 282/1445
Summary % (95% CI; I^2^)	52 (36 to 68; 85)	34 (29 to 40; 0)	13 (8 to 20; 42)		46 (36 to 56; 81)	29 (17 to 43; 85);	14 (9 to 20; 75)		50 (43 to 57; 77)	32 (23 to 42; 82)	18 (11 to 27; 90)
Strict outcome assessment‡:											
No of studies; n/N	4; 100/257	4; 91/257	-		10; 177/426	6; 71/212	-		6; 161/314	2; 34/72	-
Summary % (95% CI; I^2^)	50 (26 to 73; 91)	35 (29 to 41; 0)	-		42 (31 to 53; 78)	32 (15 to 52; 88)	-		50 (43 to 58; 40)	47 (36 to 59; 98)	-
Low risk of bias:											
No of studies; n/N	4; 73/121	3; 33/100	3; 9/100		6; 82/16	5; 45/149	6; 66/380		5; 653/1176	3; 275/1049	3; 181/1049
Summary % (95% CI; I^2^)	60 (50 to 70; 20)	33 (24 to 43; 0)	9 (4 to 15; 0)		48 (34 to 63; 68)	30 (10 to 56; 89)	17 (12 to 21; 5)		45 (33 to 58; 88)	35 (21 to 51; 89)	20 (12 to 30; 76);
Prospective studies:											
No of studies; n/N	4; 73/121	3; 33/100	3; 9/100		9; 163/390	5; 52/176	8; 81/567		5; 195/370	2; 46/164	3; 46/259
Summary % (95% CI; I^2^)	60 (50 to 70; 20)	33 (24 to 43; 0)	9 (4 to 15; 0)		42 (30 to 54; 81)	28 (10 to 50; 87)	14 (7 to 22; 80)		52 (43 to 61; 68)	27 (20 to 34; 97)	17 (10 to 27; 69)
Aged <30 years:											
No of studies; n/N	3; 63/205	3; 74/205	3; 37/205		6; 182/349	5; 63/254	6; 47/349		4; 638/1069	2; 226/938	3; 163/1033
Summary % (95% CI; I^2^)	38 (21 to 57; 76)	36 (29 to 43; 0)	18 (12 to 23; 0)		51 (40 to 63; 71)	31 (15 to 49; 82)	9 (2 to 20; 84)		60 (57 to 63; 0)	23 (20 to 26; 97)	11 (5 to 19; 67)

CIN2=cervical intraepithelial neoplasia grade 2.

* If more than one definition for regression and persistence given by authors, most stringent definition used.

† Number of studies included in analysis, number of outcomes observed/number of women attended.

‡ Only studies with strict criteria for regression (defined as normal histology and/or cytology) and persistence (defined as histological CIN2 or CIN1 and/or cytological high grade squamous cell intraepithelial lesion (HSIL), atypical squamous cells, cannot exclude HSIL (ASC-H), low grade squamous cell intraepithelial lesion (LSIL), or atypical squamous cells of unknown significance (ASC-US)).

**Fig 3:**
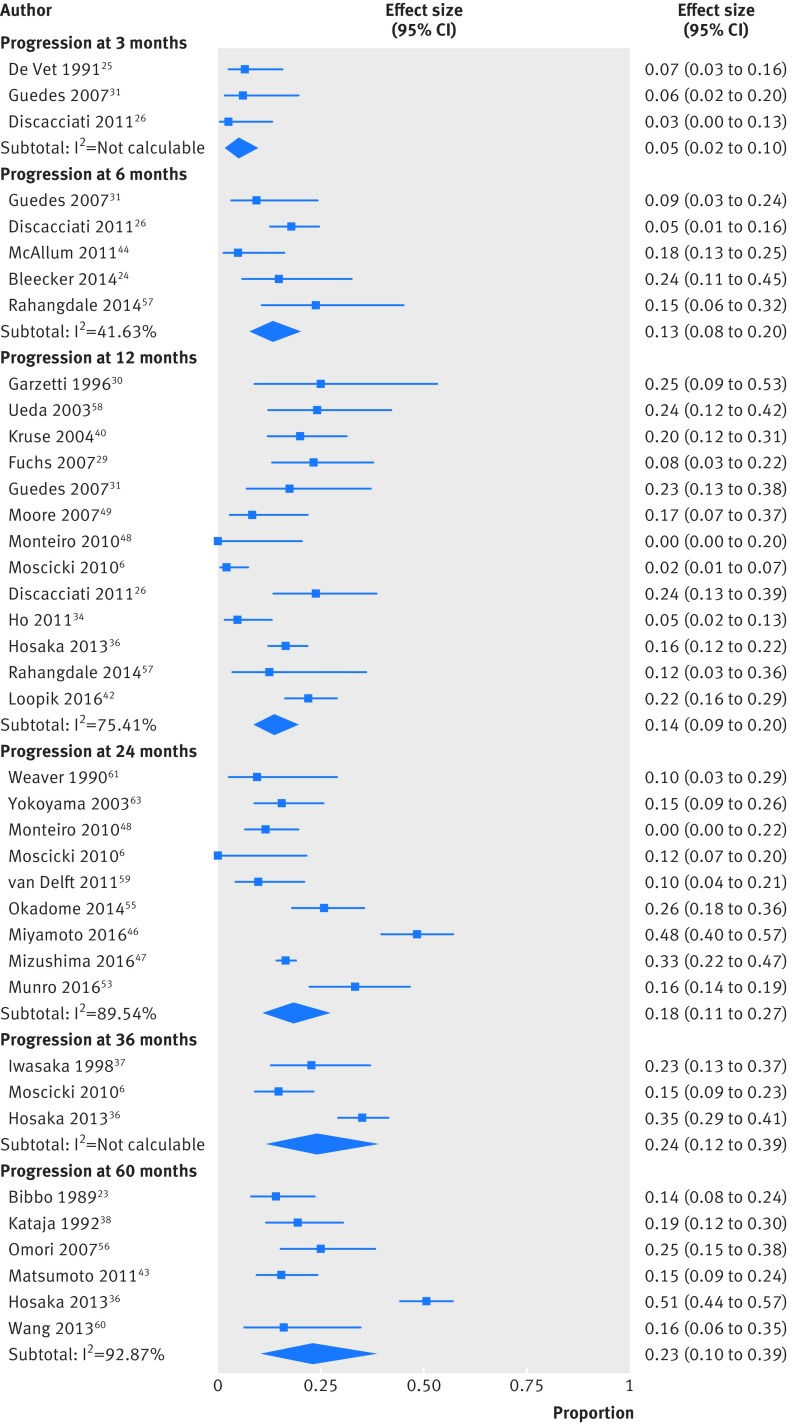
Progression rates of untreated cervical intraepithelial neoplasia grade 2 (CIN2) at different follow-up time points

The progression rate to CIN3 or worse increased with time (table 1, fig 3, and supplementary table 6). The rate was as low as 5% at three months (three studies, 7/133 women, 2% to 10%; I^2^=0%) and progressively increased from 14% at 12 months (13 studies, 131/834 women, 9% to 20%; I^2^=75%) to 18% at 24 months (nine studies, 282/1445 women, 11% to 27%; I^2^=90%) and 24% at 36 months (three studies, 105/370 women, 12% to 39%; I^2^=87%). Among the 3160 women, a total of 15 cases (0.5%, 15/3160) of cervical glandular intraepithelial neoplasia (cGIN; British Society of Colposcopy and Cervical Pathology classification—also known as adenocarcinoma in situ, AIS; Bethesda classification) were diagnosed during the follow-up period.^42^ ^53^ ^55^ Fifteen cases of invasive cervical disease occurred (0.5%, 15/3160); 13 of stage 1A1 (0.4%, 13/3160) and two of more advanced invasive disease (0.06%, 2/3160).^23^ ^36^ ^37^ ^39^

The pooled persistence rate at three months was 47% (three studies, 56/145 women, 16% to 79%; I^2^=93%). The rate decreased but remained stable at around 30% for the remaining time points (table 1, supplementary table 5): 29% (nine studies, 110/414 women, 17% to 43%; I^2^=85%) at 12 months and 32% (eight studies, 334/1257 women, 23% to 42%; I^2^=82%) at 24 months.

### Default rates

The rate of non-compliance, including missing data in retrospective studies, was 19% (five studies, 69/316 women, 7% to 35%; I^2^=88%) at six months, 15% (nine studies, 120/564 women, 6% to 25%; I^2^=87%) at 12 months, and reduced to 8% (six studies, 61/439 women, 1% to 21%; I^2^=92%) at 24 months (see supplementary table 7).

### Small study effects

Three (3/24, 13%) analyses (progression rate at 12 months and regression rates at 12 and 24 months) included 10 or more studies for visual inspection of funnel plots and the Egger’s test to have sufficient statistical power. Of these, the progression rate at 12 months showed evidence of the presence of small study effects (Egger’s test, P=0.01, 13 studies and funnel plot asymmetry).

### Sensitivity and subgroup analyses

We performed a series of sensitivity and subgroup analyses that mostly supported the results of the main analysis (see supplementary tables 4-7). By including only studies at low risk of bias, the heterogeneity continued to be high, whereas including only prospective studies and globally strict assessment of outcomes decreased heterogeneity in all outcomes but persistence. This is likely explained by varying diagnostic criteria in the literature and the temporal nature of the studied outcomes. Default rates in prospective cohort studies were around 10%, whereas in retrospective cohort studies (based on hospital registries and including potentially more women with missing data) as high as more than 20% for six months and 12 months.

The regression rates were higher and progression rates lower in women aged less than 30 years. Similarly, there was less heterogeneity with age stratification. The regression rate in women aged less than 30 was 60% at 24 months of surveillance (four studies, 638/1069 women, 57% to 63%; I^2^=0%) and peaked at 70% at 36 months (two studies, 92/131 women, 62% to 78%; I^2^=61%). The progression rate at 24 months was 11% (three studies 163/1033 women, 5% to 19%; I^2^=67%). In contrast, when we included studies in women older than 30, at 24 months the regression rate was 44% (seven studies, 181/401 women, 36% to 52%; I^2^=61%) and progression rate 23% (six studies, 119/412 women, 12% to 37%; I^2^=89%).

Women who were HrHPV and HPV16/18 negative at baseline had lower risk of progression at 24 months (3%, three studies, 1/23 women, 0% to 24%; I^2^=0%, and 5%, two studies, 1/62 women, 0% to 28%; I^2^=76%, respectively) than HrHPV or HPV16/18 positive women (25%, three studies, 38/161 women 14% to 38%; I^2^=51%, and 21%, two studies, 7/56 women, 8% to 37%; I^2^=58%, respectively). However, most of both HrHPV and HPV16/18 negative and positive women experienced regression within two years. Other studied factors in subgroup and sensitivity analyses did not noticeably affect the results.

## Discussion

Our results show that active surveillance is justified in selected women with untreated, histologically confirmed cervical intraepithelial neoplasia grade 2 (CIN2) lesions, particularly if they are young and the likelihood of compliance with follow-up is high.

Approximately half of the CIN2 lesions will regress after two years and just under one fifth will progress. In 1000 women aged less than 30 with a diagnosis of CIN2, 600 will experience regression, 230 will remain unchanged, and 110 will progress within two years of active surveillance. Out of the lesions that have more advanced disease at the end of the surveillance, the majority progress to CIN3, 5 in a 1000 have cervical glandular intraepithelial neoplasia (cGIN), and invasive cancer is rare (5 in 1000 in women of all ages; 0.6 in 1000 women if stage 1A1 is excluded). The risk of progression was particularly low in women negative for high risk human papillomavirus (HrHPV) or HPV16/18 at baseline, whereas for those who tested positive, the regression rate was 40% at two years. Adherence to follow-up was around 90% up to two years in prospective reports, which are most likely to report on true estimates. These estimates are helpful in shared decision making when deciding between active surveillance and immediate intervention.

### Strengths and limitations of this review

We conducted a systematic appraisal of the published literature on the oncological outcomes of women with untreated CIN2 lesions and the rate of compliance with surveillance. By meta-analysing the rates of progression and regression and this study provides clinicians and women current best estimates of the different patient important outcomes to assist shared decision making. The strengths of our study include the comprehensive literature search, duplicate assessment of eligibility and data abstraction, and appraisal of risk of bias. We used appropriate statistical methods to generate pooled estimates and explored possible sources of heterogeneity. We only used studies that had a histological confirmation of the initial grade of the disease, thereby decreasing the risk of misclassification bias. Although cytology has been used in some studies, we considered those to be unsuitable for inclusion as sensitivity of cytology is even lower than that of histology and the interobserver and interstudy variability are high.^64^

Our results should be interpreted with caution, however, because heterogeneity was substantial (I^2^>75%: 18/24, 75%) for most of the outcomes assessed. Although we performed a series of sensitivity and subgroup analyses, heterogeneity was still considerable, possibly as a result of the inherent difficulty in classifying lesions as CIN2. Grading of CIN, based on the thickness of the lesion in the epithelium, varies noticeably between observers.^64^ ^65^ ^66^ Misclassification of lesions affects the findings of our study and is a recognised problem in clinical practice. Here, the progression rate at six months in women aged less than 30 years was as high as 18% (three studies, 37/205 women, 12% to 23%; I^2^=0%) and was likely due to initial misclassification of a CIN3 lesion as CIN2. Despite the observed heterogeneity and even bias resulting from possible misclassification of lesions, the rates of regression were still high in young women even at the most conservative estimates.

The inclusion and exclusion criteria varied greatly across the original studies, which on the one hand might increase the applicability of the results, and on the other hand might have affected the selection of women with CIN2 lesions to be treated with active surveillance. This would therefore have introduced bias. Most of the included studies included a variety of histological grades, and only 11 out of the 36 included women purely with CIN2 lesions with prespecific inclusion and exclusion criteria. Most retrospective studies (n=13) did not have robust inclusion and exclusion criteria. Only five studies reported refusal rates. Furthermore, the description of several other factors that could have affected regression of the disease was not well documented. For instance, the use of condoms and other contraceptive techniques was reported only in some but not all studies. Only one cohort study provided data on the effect of condom use on any of the outcomes of interest of our review; condom use promoted regression in this study.^51^

Some studies provided separate outcomes on the outcome measures for all time points, whereas others only reported cumulative rates of the outcomes. Although this might have resulted in one outcome being included in more than one meta-analysis, this would not have affected the interpretation of our findings as neither the trend of the outcomes for the different follow-up time points nor the overall cumulative rates across all follow-up time points were used in the clinical interpretation of the data. For instance, the summary estimates on default rates should be interpreted with caution. The definition of non-compliance and the reporting of default rates varied greatly across studies. Only a few reported cumulative default rates at each time point. However, our estimate is likely to be true, because when we included only prospective studies, the default rates were still consistently at or below 10% and did not defer substantially from the overall rates.

Furthermore, we were unable to perform subgroup analyses according to how clinical features affect the risk of progression or regression (such as size of the lesion) as this information was not available in the included studies. Also, no randomised trials included a comparator group receiving conventional treatment with long term follow-up.

### Interpretation in light of other evidence

Our pooled rates were 46% for regression and 14% for progression at 12 months. A well cited narrative review—published a quarter of a century ago—estimated CIN2 to be more prone to progress: rates were 43% for regression and 22% for progression.^8^ However, the earlier review included neither a weighted meta-analysis nor stratification by age and length of follow-up, but estimated pooled overall proportions from a wide variety of studies dating back to the 1960s, with heterogeneous baseline and endpoint criteria. Furthermore, cytology was often used to define the grade without rigorous pathology review, and inevitably included misclassified higher or lower grade disease.^8^ Finally, as many as 30 new studies were published after the earlier review, and our comprehensive search strategy also identified four additional studies that were reported before the publication of the earlier review.^8^ All these limitations decrease certainty of these earlier estimates.

Several modelling studies applied complex mathematical frameworks to explore the clinical course of CIN.^67^ ^68^ None of these models provided data on CIN2 alone as a separate histological entity, precluding inclusion in our meta-analysis. Vink and colleagues found the 10 year incidence of cervical cancer to be 1.6% in women with a diagnosis of CIN2/3 and further stratified according to the presence of HPV16.^67^ Their finding is in line with the observed cancer incidence in our review. Van Oortmarssen and Habbema documented a regression rate for any CIN as high as 84% (95% confidence interval 76% to 92%) in women aged less than 34, whereas this was only around 40% in older women^68^; these results are also in line with our findings.

The safety of monitoring CIN2 disease has been questioned, as older reports dating a quarter of a century ago documented a risk of invasive progression as high as 5%.^8^ In our analysis, out of 3160 women only 13 (0.4%) had stage 1A1 disease and two (0.06%) more advanced disease, and most of these were diagnosed in women aged more than 30 years, although the age was not reported in three out of four studies.^23^ ^36^ ^37^ ^39^ Older age and surveillance for more than two years were common phenomena in the studies documenting invasive disease. Indeed, at least seven (47%) of these 15 cases were in women aged more than 40. A prospective cohort study from Japan (mean age 38.2, range 21-62 years) reported two cases of stage 1A1 disease,^37^ and a US retrospective cohort study reported one case after a median follow-up of five years (range 1-17 years).^23^ Neither of these studies specified the timing of diagnosis or the age of the women with cancer. One case of advanced invasive disease occurred in the placebo arm of a randomised controlled trial assessing the efficacy of oral β carotene in the treatment of CIN2/3 within two years and it is unclear whether the original histology was CIN2 or CIN3 in the case of invasion.^39^ The largest number of invasive cancers, 11 cases, was reported in a Japanese prospective study that included women with high grade squamous intraepithelial lesion (HSIL) cytology, with or without histological confirmation of CIN2 and a median follow-up of 26 months (range 1-108 months).^36^ Follow-up was based on cytological samples obtained every three months, and colposcopy and biopsy were only performed if CIN3 or worse was suspected, without description of when this was in cases of HSIL cytology. These issues, however, decrease certainty in the estimates of this Japanese study. Unlike overall rates of progression, regression, and persistence, the authors did not report all cancer cases separately based on histological confirmation of diagnosis. Ten of the 11 cancers (91%) were stage 1A1 disease and one (9%) was stage 1B1 (<2 cm). Eight (73%) were diagnosed after histologically confirmed as CIN2 lesions, seven of them in women aged more than 40, one in women aged 30-40, and none in women aged less than 30. It was unclear when the diagnoses were made during the follow-up.

In our meta-analysis, 15 (0.5%) cases of cGIN occurred among the 3160 women with CIN2. Out of these 15 cases, 14 were in women aged less than 25.^42^ ^53^ ^54^ In the retrospective cohort study from Australia with 924 expectantly managed 18-24 year old women with CIN2, eight cases (0.9%) developed cGIN within 24 months.^53^ In another retrospective cohort study from Canada with 319 women aged less than 25 at the time of CIN2 diagnosis and managed either with active surveillance or with immediate treatment, overall six cases of cGIN (1.9%) were observed within a median follow-up of 15 months.^42^ The authors did not specify whether the cases occurred in the immediate treatment group or in the surveillance group. One case of cGIN was reported in a prospective cohort study with a mean age of 37.1 (SD 6.4) years.^54^ The overall incidence of cGIN was still very low.

The benefits of active surveillance compared with treatment should outweigh the risks when conservative management is considered. Although the risk of missing glandular or invasive disease and the risk of progression is relatively low, active surveillance should be offered only to women who would likely benefit from it. The woman’s age, values and preference, wish for future pregnancies, likelihood of high compliance, as well as the clinical findings (such as the presence of a visible transformation zone) should be taken into account when conservative management is contemplated. The benefits of spontaneous regression and minimal impact on future reproduction should be balanced against the risk of non-adherence to follow-up, the risk of invasion, and the costs of repeated visits on an individual basis. The histological classification of CIN2 lesions is known to be affected by marked interobserver variability, and many CIN2 lesions are often misclassified (over-diagnosed or under-diagnosed). To minimise the risk of misclassification (of more severe abnormalities), it is recommended to discuss these cases in multidisciplinary pathology meetings on a regular basis.

Concerns could be raised about the recurrence rate of CIN lesions that are managed by active surveillance. A retrospective study by Wilkinson and colleagues has presented a follow-up after spontaneous regression of initial CIN2 in women aged less than 25 years.^69^ With a median follow-up of nearly four years after spontaneous regression, 17% developed a new high grade lesion (diagnosed as either CIN3, CIN2, cytological HSIL, or ASC-H) compared with 4% in the women whose CIN2 was primarily conventionally treated. However, the recurrence risk to a high grade abnormality after spontaneous regression of CIN2 was comparable to the 12% after conservative management of CIN1,^69^ indicating that spontaneously regressed CIN2 lesions behave like CIN1 lesions. The authors concluded that careful observation of CIN2 is an appropriate management for young women but suggested that a longer follow-up in a prospective setting would be valuable to estimate the true recurrence risk, considering as well the risk for cervical cancer being increased even after treatment of CIN2.^10^

The finding of our meta-analysis raise questions on appropriateness of the updated 2014 World Health Organization classification. The decision to simplify the previous three tiered classification system to a two tiered grouping consistent with the cytological definitions of low grade squamous intraepithelial lesion (LSIL) and HSIL arose from the appreciation of the equivocal histopathological diagnosis of CIN2 that is affected by the marked interobserver variability, which is less so in CIN3 lesions.^64^ ^65^ ^66^ The progressive potential of CIN3 has been reported to be substantially higher overall at 12%^8^ and 17% at five years.^70^ Therefore, the management of HSIL histology as a single entity might prevent more personalised care for those who are young and have lesions that are likely to regress, with substantial adverse reproductive consequences.^11^ ^12^ ^13^ ^14^ ^15^ ^16^

In a meta-analysis assessing the use of p16 protein immunostaining in cytological and histological samples, higher grade lesions stained more for p16.^71^ p16 immunostaining has been suggested by the LAST group (Lower Anogenital Squamous Terminology) as the test that could assist the pathological classification of these lesions into low grade or high grade.^72^ p16 negative lesions, formerly classified as CIN2, could be downgraded to LSIL to simplify clinical management. A study exploring the value of p16, Ki67 protein immunostaining, and HPV capsid protein L1 in improving diagnostic accuracy in cervical biopsies, found p16 to be more sensitive and less specific in diagnosing CIN2 or worse than routine pathological assessment. Ki67 did not improve the accuracy further, and HPV L1 was of no value.^73^

To date, no tests and biomarkers permit the prediction of CIN2 lesions with a true progressive potential. The results of two studies exploring the role of p16 in differentiating between regressive and progressive CIN2 were conflicting.^31^ ^56^ The first reported no predictive value, whereas the second reported value only in those strongly positive for p16. New biomolecular markers have the potential to allow the detection of CIN2 lesions with a true procarcinogeneic potential. Among many, HPV methylation and the microbiological markers have been shown in cross sectional studies to correlate to disease severity, and data on serial longitudinal samples are still awaited.^74^ ^75^ ^76^ ^77^ ^78^

### Conclusion

The results of our analysis show higher rates of regression and lower rates of progression of histologically confirmed CIN2 lesions than previously reported, particularly in women aged less than 30. Conservative management with active surveillance, instead of immediate local excision, is therefore justified in selected women, especially if further pregnancies are considered and compliance with surveillance is likely to be high (primum non nocere). With increasing maternal age and increasing awareness that local treatment for CIN is associated with increased preterm birth and mid-trimester loss,^11^ ^12^ ^13^ ^14^ ^15^ ^16^ treating only those with disease that has a true progressive potential is of utmost importance. In cases of disease that persists beyond two years, treatment is likely to be warranted.

**What is already known on this topic**

The clinical course of cervical intraepithelial neoplasia grade 2 (CIN2) is not well established
CIN2 on a colposcopically directed cervical biopsy has been considered the clinical cut-off to proceed to treatment
Some studies, however, have suggested that spontaneous regression rates may be high; this could be especially important for women of reproductive age because local treatments can be harmful for future pregnancies

**What this study adds**

Half of untreated CIN2 lesions (50%) regress spontaneously and one in five (18%) progress to CIN3 or worse within two years of surveillance—the rates are 60% and 11% in women aged less than 30
Among more than 3000 women, there were only 13 stage 1A1 (0.4%) and two more advanced (0.06%) invasive cases, most in women older than 30
Active surveillance of CIN2 rather than immediate intervention is justified, especially among younger women

## Web extra

Extra material supplied by authors

**Supplementary information:** additional information supplied by authors

## Notes

Contributors: KT, IK, PN, MK, and KAOT conceived and designed the study. KT, AA, RA, LCH, SGL, MJ, KJ, MKi, KL, SO, RT, SV, PN, and IK screened the articles. KT, AA, and IK collated the data. KT, AA, KAOT, MK, and IK interpreted the data. All authors drafted and revised critically the manuscript for important intellectual content. All authors gave final approval of the version to be published and have contributed to the manuscript. IK is the guarantor. MK and IK are joint last senior authors.

Ethical approval: Not required.

Data sharing: No additional data available.

Transparency: The lead author (IK) affirms that the manuscript is an honest, accurate, and transparent account of the study being reported; that no important aspects of the study have been omitted; and that any discrepancies from the study as planned have been explained.
