# Supplementary material for: Clinical course of untreated cervical intraepithelial neoplasia grade 2 under active surveillance: systematic review and meta-analysis
Source: BMJ. 2018 Feb 27;360:k499. doi: 10.1136/bmj.k499 (PMC5826010; doi:10.1136/bmj.k499)
Supplement: Supplementary file 1 — Supplementary information: additional information supplied by authors [file taik040043.ww1.pdf]

Supplementary material

Supplementary methods

Search strategies for databases in the systematic review of CIN 2 natural history

Medline Ovid

1. Uterine Cervical Neoplasms/ or Cervical Intraepithelial Neoplasia/
2. CIN 2.mp.
3. CIN2.mp.
4. CIN grade 2.mp.
5. cervical intraepithelial neoplasia grade 2.mp.
6. dysplasia moderata.mp.
7. moderate dysplasia.mp.
8. moderate dysplasia of the uterine cervix.mp.
9. moderate cervical dysplasia.mp.
10. intermediate dysplasia.mp.
11. moderate cervical intraepithelial lesion.mp.
12. Neoplasm Regression, Spontaneous/
13. Disease Progression/
14. prognosis.mp.
15. prognoses.mp.
16. outcome.mp.
17. courses.mp.
18. forecast of outcome.mp.
19. determination of prognosis.mp.
20. outcome prediction.mp.
21. progression.mp.
22. regression.mp.
23. clearance.mp.
24. persistence.mp.
25. persistence.mp.
26. recurrence.mp.
27. natural history.mp.
28. untreated.mp.
29. watchful waiting.mp.
30. expectant management.mp.
31. active surveillance.mp.
32. survival analysis.mp.
33. follow-up studies.mp.
34. follow-up.mp.
35. mortality.mp.
36. survival.mp.
37. morbidity.mp.
38. treatment.mp.
39. management.mp.
40. incidence.mp.
41. prevalence.mp.
42. 2 or 3 or 4 or 5 or 6 or 7 or 8 or 9 or 10 or 11
43. 14 or 15 or 16 or 17 or 18 or 19 or 20 or 21 or 22 or 23 or 24 or 25 or 26 or 27 or 28 or 29 or 30 or 31 or 32 or 33 or 34 or 35 or 36 or 37 or 38 or 39 or 40 or 41
44. 12 or 13
45. 1 or 42
46. 43 or 44
47. 45 and 46
48. limit 47 to yr="1973 - 2013"

Key: mp=title, abstract, original title, name of substance word, subject heading word, keyword heading word, protocol supplementary concept word, rare disease supplementary concept word, unique identifier

A second otherwise identical search 2014 onward was performed without MeSH terms (Uterine Cervical Neoplasms/ or Cervical Intraepithelial Neoplasia/ and Neoplasm Regression, Spontaneous/ or Disease Progression/).

Embase via Scopus

((TITLE-ABS-KEY (outcome)) OR (TITLE-ABS-KEY (prognoses)) OR (TITLE-ABS-KEY (prognosis)) OR (TITLE-ABS-KEY (follow-up)) OR (TITLE-ABS-KEY (mortality)) OR (TITLE-ABS-KEY (survival)) OR (TITLE-ABS-KEY (morbidity)) OR (TITLE-ABS-KEY (clearance)) OR (TITLE-ABS-KEY (regression)) OR (TITLE-ABS-KEY (progression)) OR (TITLE-ABS-KEY (determination of prognosis)) OR (TITLE-ABS-KEY (outcome prediction)) OR (TITLE-ABS-KEY (forecast of outcome)) OR (TITLE-ABS-KEY (courses)) OR (TITLE-ABS-KEY (survival analysis)) OR (TITLE-ABS-KEY (active surveillance)) OR (TITLE-ABS-KEY (expectant management)) OR (TITLE-ABS-KEY (watchful waiting)) OR (TITLE-ABS-KEY (untreated)) OR (TITLE-ABS-KEY (natural history)) OR (TITLE-ABS-KEY (recurrence)) OR (TITLE-ABS-KEY (persistence)) OR (TITLE-ABS-KEY (treatment)) OR (TITLE-ABS-KEY (management)) OR (TITLE-ABS-KEY (incidence)) OR (TITLE-ABS-KEY (prevalence)) OR (KEY (disease progression)) OR (KEY (neoplasm regression,spontaneous)) AND (TITLE-ABS-KEY (cervical intraepithelial neoplasia grade 2)) OR (TITLE-ABS-KEY (cin2)) OR (TITLE-ABS-KEY (cin 2)) OR (TITLE-ABS-KEY (intermediate dysplasia)) OR (TITLE-ABS-KEY (moderate cervical intraepithelial lesion)) OR (TITLE-ABS-KEY (moderate cervical dysplasia)) OR (TITLE-ABS-KEY (moderate dysplasia of the uterine cervix)) OR (TITLE-ABS-KEY (moderate dysplasia)) OR (TITLE-ABS-KEY (dysplasia moderata)) OR (TITLE-ABS-KEY (cin grade 2)) OR (KEY (uterine cervical neoplasms)) OR (KEY (cervical intraepithelial neoplasia)) AND NOT INDEX (medline)) AND INDEX (embase)

Cumulative Index to Nursing and Allied Health Literature (CINAHL)

((MH "Prognosis") OR (MH "Treatment Outcomes") OR (MH "Neoplasm Regression, Spontaneous")
OR (MH "Disease Progression")
OR TX prevalence
OR incidence
OR management
OR treatment
OR morbidity
OR survival
OR mortality
OR follow-up
OR TX watchful waiting
OR TX active surveillance
OR TX expectant management
OR TX untreated
OR TX natural history
OR TX recurrence
OR TX persistence
OR X clearance
OR TX regression
OR TX progression
OR TX courses
OT TX outcome
OR TX prognoses
OR TX determination of prognosis)
AND (TX intermediate dysplasia

OR TX moderate cervical intraepithelial lesion
OR TX moderate cervical dysplasia
OR TX moderate dysplasia of the uterine cervix
OR TX moderate dysplasia
OR TX cervical intraepithelial neoplasia grade 2
OR TX CIN grade 2
OR TX CIN 2
OR TX CIN2
OR (MH "Cervix Neoplasms") OR (MH "Cervical Intraepithelial Neoplasia"))

Supplementary table 1 – Risk of bias definitions

Domain	Low risk of bias	High risk of bias
Assessment of exposure	Secure record (e.g. hospital records)	Structured interview, self-written report
Presence of outcome at start of study (confirmation of initial diagnosis CIN2)	Histological confirmation	Confirmation by cytology, colposcopic impression and/or HPV testing
Assessment of outcome	Outcome confirmed with multiple methods including histology	Outcome confirmed only with cytology, colposcopy or HPV testing
Loss to follow-up	Loss to follow-up less than 10%	Loss to follow-up over 20% or not adequately reported
Representativeness of cohort	All eligible (CIN2) cases in a predefined time period and population included	Not fulfilling low risk criteria<br>Predefined age range is not considered high risk of bias

Supplementary table 2. Characteristics of included studies.

Author, year	Country	Study design	N followed-up [1]	Median age in y (range) [2]	Definition of regression	Definition of persistence	Definition of progression	F-u protocol [3]	Definition of default	Time points included [4]	HPV DNA test (HPV types) [5]
Alvarez, 2003	USA	RCT	15	24.5 (18-40)	lenient: ≤CIN1	lenient: CIN2	CIN3+	Cyto&colpo every 1.5m; LLETZ at 3m	Not completing the study per protocol specifications	3m	NA
Bibbo, 1989	USA	Retrospective cohort	78 [1]	NA	strict: negative histology or cyto for ≥12m	strict: CIN1/2 after 12m	CIN3+	No specific protocol	F-u<1y	60m	NA
Bleecker, 2014	USA	Retrospective cohort	46	23 (16-30)	lenient: ≤CIN1	lenient: CIN2	CIN3+	Colpo+/-cyto every 6m for up to 2y	Not returning for f-u after exclusion of women who had planned f-u elsewhere, or no endpoint biopsy	6m	NA
de Vet, 1991 [6]	the Netherlands	RCT	61 [1]	NA (20-65)	lenient: ≤CIN1 or PAP I-IIIA	lenient: CIN2	CIN3+	Cyto/colpo at 3m; biopsy at doctor's discretion (but preferable)	Withdrawal of consent, missed f-u, other treatment, pregnancy	3m	NA
Discacciati, 2011	Brazil	Prospective cohort	50	25 (17-47)	strict: normal histology (if available), cyto and colpo	strict: abnormal histology, cyto, or colpo (at 3m/6m); histological confirmation (CIN1/2) at 12m	CIN3+	Cyto&colpo every 3m for 12m; biopsy when CIN3+ was suspected	Missed f-u or discontinued (pregnancy, HIV, missed 3 consecutive f-u, persistent CIN2 at some visit but did not attend f-u at 12m)	3m, 6m, 12m	PCR (HR, 16)

Fuchs, 2007	USA	Retrospective cohort	40	NA (≤21)	strict: normal colpo (+/- histology) + ≥2 normal smears; ≥3 consecutive normal smears if no colpo	strict: not meeting criteria for regression or progression	CIN3+	Colpo at 4-6m (not all women); intervals of cyto not reported	Not returning for repeat evaluation	12m, 24m, 36m	NA
Garzetti, 1996	Italy	Prospective cohort	12	34 (22-41)	strict: normal histology, colpo and negative HPV DNA test	strict: Abnormal histology, colpo, or positive HPV DNA test	CIN3+	HPV DNA test (PCR)/cyto/colpo at 3, 6 and 12m; biopsy at 12m	No woman is reported to have been lost to f-u after consent	12m	NA
Guedes, 2010	Brazil	Prospective cohort	45	30 (18-61) [2]	strict: normal histology or cyto lenient: ≤CIN1 or ≤LSIL	strict: CIN1/2 or ASCUS/LSIL lenient: CIN2	CIN3+	Cyto&colpo every 3m for 12m; biopsy when progression was suspected; LLETZ if abnormal colpo at 12m	Withdrawal of initial consent	3m, 6m, 12m	NA
Hillemanns, 2015	USA + 4 European countries	RCT	21	27 (18-60)	lenient: ≤CIN1 and ≤LSIL at 3m; ≤LSIL at 6m	lenient: CIN2 or ASCH/HSIL	CIN3+	Colpo&biopsy at 3m; cyto/HPV DNA test (PCR) at 3m and 6m	Withdrawal of consent, lost to f-u, withdrawal (by the gynaecologist, due to non-compliance, or for other causes, such as pregnancy and progression to CIN3)	3m, 6m	NA

Ho, 2011	USA	Prospective cohort	63 [1]	29.8 (≥18) [2]	strict: no CIN<br>lenient: ≤CIN1	strict: CIN1/2<br>lenient: CIN2	CIN3+	Cyto/colpo/HPV DNA test (PCR and/or Southern blot)/blood sampling every 3m for 12m; biopsy at 12m	Not completing the 12m visit, not sufficient biopsy material	12m	NA
Hording, 1991	Denmark	Prospective cohort	14	28 (21-69)	strict: normal colpo + 3 normal smears	strict: not fulfilling regression criteria with histological confirmation	CIN3+	Cyto&colpo every 3m	No woman is reported to have been lost to f-u after consent	12m	ISH (16, 18)
Hosaka, 2013	Japan	Prospective cohort	231	progression: 44 (23-77)	strict: 2 normal smears	strict: abnormal cyto	CIN3+	Cyto every 3m (baseline cyto was HSIL in all included women); colposcopically-directed biopsy when CIN3+ was suspected	Lost to f-u	12m, 36m, 60m	-
Iwasaka, 1998	Japan	Prospective cohort	44	38.2 (21-62) [2]	strict: negative histology, cyto and colpo	strict: not meeting criteria for regression or progression	CIN3+	Cyto&colpo every 3m; biopsy when CIN3+ was suspected	No woman is reported to have been lost to f-u after consent	36m	NA
Kataja, 1992	Finland	Prospective cohort	67 [1]	28.6 (15-66) [2]	strict: negative histology, cyto and colpo	strict: abnormal histology, cyto, or colpo	CIN3+	Cyto&colpo every 6m; biopsy whenever cytology indicated HPV or CIN	NR	60m	NA

Keefe, 2001	USA	RCT	20 [1]	29.8 (18-54.6) [2]	strict: no CIN	strict: CIN1/2	CIN3+	Cyto&colpo every 3m for 24m	Withdrawal, moving, pregnancy, lost to f-u, non-compliance	24m	NA
Kruse, 2004	Norway	Prospective cohort	65 [1]	progression: 39.7 non – progression: 35.6 [2]	NA; non-progression: negative histology or cyto	NA	CIN3+	NR	F-u <4m, lost to f-u, Ki67 immunoquantitation not possible	12m, 36m	NA
Loopik, 2016	Canada	Retrospective cohort	211 [1]	NA (<25)	lenient: ≤CIN1	strict: CIN1/2	CIN3+	Colpo/cyto but no other details; endpoint biopsy or treatment >6m after diagnosis of CIN2	Endpoint biopsy not done/unknown	12m	NA
Matsumoto, 2011	Japan	Prospective cohort	91 [1]	36 (18-54) [2]	strict: normal colpo + ≥2 normal smears	strict: not meeting criteria for regression or progression	CIN3+	Cyto&colpo every 3-4m; biopsy when progression to CIN3+ was suspected: HSIL smear and major colposcopic changes	Insufficient f-u (<2 f-u visits)	24m, 60m	PCR (HR, 16, 18)
McAllum, 2011 [6]	New Zealand	Retrospective cohort	157	20.9 (<25) [2]	strict: no CIN lenient: ≤CIN1	strict: CIN1/2 lenient: CIN2	CIN3+	NR (management was based on local practice, and treatment decisions were the responsibility of the individual colposcopist)	No endpoint biopsy	6m, 12m, 24m	NA

Meyskens, 1994	USA	RCT	66 [1]	NA	strict: negative histology and cyto	strict: abnormal histology or cyto	CIN3+	Cyto&colpo at 9, 12, 15, 21 and 27m; biopsy at 15m (at 9 or 12m if severe disease; at 21 or 27m if woman did not attend f-u at 15m)	Lost to f-u/no f-u biopsy	12m	NA
Miyamoto, 2016	Japan	Retrospective cohort	122 [1]	regression: 39.8 (NA) persistence: 36.5 (NA) progression: 37 (NA) [2]	strict: ≥2 normal smears for ≥24m	strict: no change on histology and colpo	CIN3+ or colpo suggestive of CIN3+ after ≥6m	Colpo&cyto every 3m; biopsy if any abnormal lesions were suspected	Women without 2 years' f-u information	24m, 36m	PCR (HR)
Mizushima, 2016	Japan	Retrospective cohort	54	38.6 (NA) [2]	lenient: ≤CIN1 or 3 normal smears	lenient: not meeting criteria for regression or progression	CIN3+	Colpo&cyto every 3-4m; biopsy routinely once a year for cases with abnormal colpo and when novel abnormal findings were detected	No woman is reported to have been lost to f-u after consent	24m	NA
Monteiro, 2010	Brazil	Retrospective (mostly) + prospective cohort	17	15.3 (11-19) [2]	NA	NA	CIN3+	Cyto every 6m for 2y	F-u<12m; women with interrupted f-u during 12-24m were considered losses for analysis at 24m but were included in	12m, 24m	NA

									analysis at 12m		
Moore, 2007	USA	Retrospective cohort	55	19 (13-21)	lenient: ≤CIN1 or less severe cyto than baseline	lenient: not meeting criteria for regression or progression	CIN3+ or more severe cyto than baseline	Colpo&cyto every 4-6m; biopsy if indicated to rule out CIN3+	NR	12m	NA
Moscicki, 2010	USA	Prospective cohort	120	20.3 (13-24)	strict: 3 normal smears (+ normal biopsy if obtained)	strict: not meeting criteria for regression or progression	CIN3+	Colpo/cyto every 4m; biopsy when CIN3+ was suspected	Verbal refusal to enter f-u; no return for f-u	12m, 24m, 36m	PCR (16, 18)
Munk, 2012	Norway	Prospective cohort	39	30.8 (25-41)	lenient: ≤CIN1	lenient: CIN2	CIN3+	1st visit (0w): colpo, biopsy, endocervical curettage; 2nd visit (7-9w): colpo; 3rd visit (12-24w): colpo, LLETZ	No woman is reported to have been lost to f-u	3m	PCR (16)
Munro, 2016	Australia	Retrospective cohort	924 [1]	NA (18-24)	lenient: ≤CIN1 or ≤LSIL	lenient: CIN2 or cyto indicating CIN2	CIN3+ or smears indicating CIN3+	NR	F-u<12m	24m	NA
Okadome, 2014	Japan	Prospective cohort	122	37.1 (NA) [2]	lenient: ≤CIN1	lenient: CIN2	CIN3+	Colpo, cyto& HPV DNA test every 6m for 2y; biopsy at 24m or when CIN3+ was suspected	Disagreement on progression diagnosis; pregnancy; relocation; lost to f-u; difficulty in attending the hospital; other	24m	PCR (HR, 16)

Omori, 2007	Japan	Prospective cohort	52	regression: 36.1 (19-64) persistence: 34.5 (20-47) progression: 39.2 (28-51) [2]	strict: normal cyto/colpo	strict: abnormal cyto/colpo for >2y	CIN3+	Colpo&cyto every 3-4m; biopsy when CIN3+ was suspected	No woman is reported to have been lost to f-u	36m, 60m	ISH (HR)
Rahangdale, 2014	USA	RCT	29	23 (18-29)	strict: no CIN lenient: ≤CIN1	strict: CIN1/2 lenient: CIN2	CIN3+	Colpo, cyto & biopsy at 6 and 12m; no biopsy was taken at 12m if normal f-u at 6m and normal colpo at 12m	Lost to f-u or deferred biopsy	6m, 12m	NA
Ueda, 2003	Japan	Retrospective cohort + case control (for HPV)	29 [1]	NA	NA	NA	CIN3+	Colpo&cyto every 1-3m; biopsy for histological confirmation of colposcopically visible lesions	F-u<6m	6m, 12m, 24m	PCR (HR, 16)
van Delft, 2011	the Netherlands	Retrospective cohort	55	39 (18-84)	strict: normal cyto	strict: abnormal cyto	CIN3+	Cyto; at least one colpo and biopsy during f-u	Lost to f-u	24m	NA
Wang, 2013	China	Prospective cohort	25 [1]	39 (35-45) [2]	strict: no CIN lenient: ≤CIN1	strict: CIN1/2 lenient: CIN2	CIN3+	Cyto, HPV DNA test, colpo if ASCUS with HR-HPV (+) or LSIL and worse /biopsy of lesions found during	Lost to f-u; death; hysterectomy for non-cervical disease; treatment for benign cervical	60m	PCR (HR)

								colpo/endocervical curettage if squamocolumnar junction not visible	disease; incomplete data; inconclusive diagnosis		
Weaver, 1990	USA	Retrospective cohort	21 [1]	27.1 (19-36) [2]	strict: ≥2 normal smears (+ normal histology if performed)	strict: ≥2 abnormal smears (or abnormal histology if performed)	CIN3+ or ≥2 smears indicating CIN3+	A minimum of 2 satisfactory cervical cyto examinations, one of which was performed at least 1 year after histological diagnosis of CIN2	F-u<12m	24m	ISH (HR, 16, 18)
Woodman, 1993	UK	RCT	39	33 (20-65)	strict: 2 normal smears	strict: abnormal cyto without histological progression to CIN3+	CIN3+	Colpo&cyto every 4m; biopsy when CIN3+ was suspected	Lost to f-u	12m	NA
Yokoyama, 2003	Japan	Prospective cohort	71 [1]	NA (≤55)	NA	NA	CIN3+	Colpo&cyto every 4m; biopsy when progression or regression was suspected	F-u<3m	24m	NA

Abbreviations: ASCH: atypical squamous cells-cannot exclude HSIL; ASCUS, atypical squamous cells of undetermined significance; CIN, cervical intraepithelial neoplasia; colpo, colposcopy; cyto, cytology; f-u, follow-up; HR, all high-risk HPV-types altogether; HSIL, high-grade squamous intraepithelial lesion; ISH, in-situ hybridization; LLETZ, large loop excision of the transformation zone; LSIL, low-grade squamous intraepithelial lesion; m, month; NA, data not available; NR, not reported; PAP, Papanicolaou grade (I to V); PCR, polymerase chain reaction; RCT, randomized controlled trial; w, weeks; y, years

[1] Some studies do not state how many women with CIN2 observed expectantly entered follow-up. In those cases, N of women completing follow-up is reported
[2] Some studies do not report the median but only the mean

[3] In some studies, the criteria for biopsy were not explicitly reported; in these studies, we don't report biopsy in f-u protocol but we can assume that biopsies were taken at least when progression to CIN3+ was suspected

[4] The time points reported are not the exact ones. Instead, they are compromises we had to make in order to perform the meta-analysis (i.e. if the median/mean f-u was 15m, we considered the time point 12m)

[5] Inside the parentheses we report the baseline HPV types for which the subsequent regression/persistence/progression rates at 24m are given. We report only the HPV types that we used in our meta-analysis (i.e. high-risk (HR), 16 and 18)

[6] Data extracted with different criteria for regression, persistence and progression than those originally used by the authors

Supplementary Table 3: Risk of bias assessment.

Criteria used: 1=Definitely yes; 2=Probably yes; 3=Probably no; 4=Definitely no.

Author, year	Assessment of exposure [1]	Outcome present at start [2]	Assessment of outcome [3]	Loss to follow-up [4]	Representativeness of cohort [5]	Low risk of bias [6]
Alvarez, 2003	1	1	1	2	1	Yes
Bibbo, 1989	1	1	2	3	2	No
Bleecker, 2014	1	1	1	4	2	No
de Vet, 1991	1	1	2	1	1	Yes
Discacciati, 2011	1	1	2	2	1	Yes
Fuchs, 2007	1	1	2	2	1	Yes
Garzetti, 1996	1	1	2	1	1	Yes
Guedes, 2010	1	1	2	1	1	Yes
Hillemanns, 2015	1	1	2	2	1	Yes
Ho, 2011	1	1	1	3	2	No
Hording, 1991	1	1	2	2	1	Yes
Hosaka, 2013	1	1	2	1	1	Yes
Iwasaka, 1998	1	1	2	1	1	Yes
Kataja, 1992	1	1	2	2	1	Yes
Keefe, 2001	1	1	1	4	2	No
Kruse, 2004	1	1	1	3	2	No
Loopik, 2016	1	1	3	1	1	No
Matsumoto, 2011	1	1	2	2	1	Yes
McAllum, 2011	1	1	1	3	1	No
Meyskens, 1994	1	1	1	4	2	No
Miyamoto, 2016	1	1	2	3	2	No
Mizushima, 2016	1	1	2	1	1	Yes
Monteiro, 2010	1	1	3	2	1	No
Moore, 2007	1	1	2	4	2	No
Moscicki, 2010	1	1	2	3	1	No
Munk, 2012	1	1	1	1	1	Yes
Munro, 2016	1	1	2	2	1	Yes
Okadome, 2014	1	1	1	3	2	No
Omori, 2007	1	1	2	1	1	Yes
Rahangdale, 2014	1	1	1	2	1	Yes
Ueda, 2003	1	1	1	3	2	No
van Delft, 2011	1	1	3	1	1	No
Wang, 2013	1	1	1	4	2	No
Weaver, 1990	1	1	3	3	2	No
Woodman, 1993	1	1	3	1	1	No
Yokoyama, 2003	1	1	2	1	1	Yes

[1] Only secure records (e.g. hospital records, trial records etc.) were used
[2] Only histological confirmation of CIN2 at the beginning of the study
[3] 1=Histology was used along with other methods; 2=Cytology and colposcopy were used (usually also histology when progression was suspected); 3=Only histology was used (colposcopy only when deemed necessary); 4=Only cytology, only colposcopy, or only HPV test
[4] Low risk=loss to follow-up <10%; medium risk=loss to follow-up 10-20%; high risk=loss to follow-up >20% or not reported adequately
[5] Low risk=all eligible cases in the defined setting over the defined period; high risk=not fulfilling criteria for low risk
[6] Yes=only 1 or 2 in all modalities of bias risk assessment; no=at least one 3 or 4 in any modality of bias risk assessment.

Supplementary table 4. Regression rates for CIN 2 in all time points and subgroup and sensitivity analyses.

		Regression 3 months	Regression 6 months	Regression 12 months	Regression 24 months	Regression 36 months	Regression 60 months
**Main analysis** [3]	**N of studies n/N** [1]	6<br>97/208	7<br>139/328	13<br>300/628	11<br>819/1470	6<br>194/414	3<br>70/170
	**Summary % (95% CI; I^2)** [2]	42<br>(24-61; 86)	52<br>(36-68; 85)	46<br>(36-56; 81)	50<br>(43-57; 77)	50<br>(33-68; 92)	44<br>(24-66; 86)
**Lenient criteria** [4]	**N of studies n/N**	6<br>115/208	7<br>172/328	13<br>325/628	11<br>819/1470	6<br>194/414	3<br>76/170
	**Summary % (95% CI; I^2)**	52<br>(31-72; 88)	57<br>(47-66; 59)	50<br>(39-62; 86)	50<br>(43-57; 77)	50<br>(33-68; 92)	52<br>(24-80; 93)
**Exact follow-up only** [5]	**N of studies n/N**	4<br>76/136	4<br>62/110	7<br>140/286	4<br>135/222	1<br>65/95	NA
	**Summary % (95% CI; I^2)**	49<br>(25-73; 87)	56<br>(47-66; 0)	50<br>(38-62; 69)	61<br>(54-68; 0)	68<br>(59-77; NE)	NA
**Lenient assesment of outcome** [6]	**N of studies n/N**	5<br>100/169	6<br>146/287	6<br>210/324	5<br>658/1156	NA	1<br>20/25
	**Summary % (95% CI; I^2)**	54<br>(30-77; 89)	56<br>(45-66; 60)	66<br>(59-72; 12)	50<br>(37-63; 87)	NA	80<br>(61-91; NE)
**Strict assessment of outcome** [7]	**N of studies n/N**	2<br>24/72	4<br>100/257	10<br>177/426	6<br>161/314	6<br>194/414	3<br>70/170
	**Summary % (95% CI; I^2)**	33<br>(23-45; 99)	50<br>(26-73; 91)	42<br>(31-53; 78)	50<br>(43-58; 40)	50<br>(33-68; 92)	44<br>(24-66; 86)
**Low risk of bias**	**N of studies n/N**	6<br>97/208	4<br>73/121	6<br>82/163	5<br>653/1176	3<br>71/132	1<br>36/67
	**Summary % (95% CI; I^2)**	42<br>(24-61; 86)	60<br>(50-70; 20)	48<br>(34-63; 68)	45<br>(33-58; 88)	55<br>(34-75; 83)	54<br>(42-65; NE)

		Regression 3 months	Regression 6 months	Regression 12 months	Regression 24 months	Regression 36 months	Regression 60 months
**High risk of bias**	**N of studies n/N**	NA	3 66/207	7 218/465	6 166/294	3 123/282	2 34/103
	**Summary % (95% CI; I^2)**	NA	41 (21-63; 83)	44 (31-58; 87)	55 (47-64; 42)	45 (19-74; 96)	32 (24-42; 98)
**Only European studies**	**N of studies n/N**	2 58/100	NA	4 34/131	1 22/51	1 30/65	1 36/67
	**Summary % (95% CI; I^2)**	59 (49-68; 98)	NA	25 (17-34; 7)	43 (31-57; NE)	46 (35-58; NE)	54 (42-65; NE)
**Only North America**	**N of studies n/N**	1 3/15	2 23/48	6 208/397	4 97/172	2 92/131	1 20/78
	**Summary % (95% CI; I^2)**	20 (7-45; NE)	48 (34-62; 99)	52 (41-62; 71)	55 (45-64; 29)	70 (62-78; 76)	26 (17-36; NE)
**Only South America**	**N of studies n/N**	2 24/72	2 48/73	3 58/100	1 10/14	NA	NA
	**Summary % (95% CI; I^2)**	33 (23-45; 96)	66 (54-76; 96)	58 (37-77; 75)	71 (45-88; NE)	NA	NA
**Only Asia**	**N of studies n/N**	NA	1 15/29	NA	4 140/309	3 72/218	1 14/25
	**Summary % (95% CI; I^2)**	NA	52 (34-69; NE)	NA	43 (31-56; 80)	37 (19-57; 87)	56 (37-73; NE)
**North America excluded**	**N of studies n/N**	5 94/193	5 116/280	7 92/231	7 722/1298	4 102/283	2 50/92
	**Summary % (95% CI; I^2)**	46 (26-66; 88)	53 (32-74; 90)	40 (24-57; 83)	49 (39-58; 85)	39 (25-54; 85)	54 (44-65; 95)
**Europe excluded**	**N of studies n/N**	4 39/108	7 139/328	9 266/497	10 797/1419	5 164/349	2 34/103
	**Summary % (95% CI; I^2)**	36 (22-50; 54)	52 (36-68; 85)	54 (45-62; 69)	51 (43-58; 77)	51 (30-72; 94)	32 (24-42; 98)

		Regression 3 months	Regression 6 months	Regression 12 months	Regression 24 months	Regression 36 months	Regression 60 months
**Only 1980's**	**N of studies n/N**	1<br>46/61	NA	3<br>31/119	1<br>9/21	NA	2<br>56/145
	**Summary % (95% CI; I^2)**	75<br>(63-84; NE)	NA	26<br>(16-38; 38)	43<br>(24-63; NE)	NA	38<br>(30-46; 69)
**Only 1990's**	**N of studies n/N**	1<br>3/15	1<br>15/29	2<br>37/75	2<br>38/91	1<br>16/44	NA
	**Summary % (95% CI; I^2)**	20<br>(7-45; NE)	52<br>(34-69; NE)	49<br>(38-61; 90)	42<br>(31-52; 90)	36<br>(24-51; NE)	NA
**Only 2000's**	**N of studies n/N**	3<br>36/111	3<br>88/230	6<br>123/254	7<br>757/1304	4<br>148/305	1<br>14/25
	**Summary % (95% CI; I^2)**	32<br>(24-42; 0)	52<br>(21-81; 94)	52<br>(38-65; 75)	56<br>(50-62; 55)	55<br>(28-79; 95)	56<br>(37-73; NE)
**Only 2010's**	**N of studies n/N**	NA	2<br>23/48	2<br>109/180	1<br>15/54	NA	NA
	**Summary % (95% CI; I^2)**	NA	48<br>(34-62; 95)	61<br>(53-68; 95)	28<br>(18-41; NE)	NA	NA
**2000's excluded**	**N of studies n/N**	3<br>61/97	4<br>51/98	7<br>177/374	4<br>62/166	2<br>46/109	2<br>56/145
	**Summary % (95% CI; I^2)**	53<br>(22-83; 88)	52<br>(42-62; 0)	41<br>(26-56; 86)	37<br>(29-47; 23)	42<br>(33-52; 74)	38<br>(30-46; 74)
**Only retrospective cohorts**	**N of studies n/N**	NA	3<br>66/207	4<br>137/238	6<br>624/1100	2<br>55/158	1<br>20/78
	**Summary % (95% CI; I^2)**	NA	41<br>(21-63; 83)	55<br>(43-67; 53)	48<br>(35-61; 83)	34<br>(27-42; 97)	26<br>(17-36; NE)
**Only prospective studies**	**N of studies n/N**	6<br>97/208	4<br>73/121	9<br>163/390	5<br>195/370	4<br>139/256	2<br>50/92
	**Summary % (95% CI; I^2)**	42<br>(24-61; 86)	60<br>(50-70; 20)	42<br>(30-54; 81)	52<br>(43-61; 68)	52<br>(38-66; 80)	54<br>(44-65; 93)

		Regression 3 months	Regression 6 months	Regression 12 months	Regression 24 months	Regression 36 months	Regression 60 months
**Only RCTs**	**N of studies n/N**	3<br>61/97	2<br>25/48	3<br>34/121	1<br>10/20	NA	NA
	**Summary % (95% CI; I^2)**	53<br>(22-83; 88)	52<br>(38-66; 94)	30<br>(15-49; 73)	50<br>(30-70; NE)	NA	NA
**Only prospective cohorts**	**N of studies n/N**	3<br>36/111	2<br>48/73	6<br>129/269	4<br>185/350	4<br>139/256	2<br>50/92
	**Summary % (95% CI; I^2)**	32<br>(24-42; 0)	66<br>(54-76; 3)	48<br>(35-61; 74)	52<br>(42-63; 76)	52<br>(38-66; 80)	54<br>(44-65; 93)
**Upper limit of age range 30 or younger**	**N of studies n/N**	NA	3<br>63/205	6<br>182/349	4<br>638/1069	2<br>92/131	NA
	**Summary % (95% CI; I^2)**	NA	38<br>(21-57; 76)	51<br>(40-63; 71)	60<br>(57-63; 0)	70<br>(62-78; 61)	NA
**Upper limit of age range >30**	**N of studies n/N**	6<br>97/208	4<br>76/123	7<br>118/279	7<br>181/401	4<br>102/283	3<br>70/170
	**Summary % (95% CI; I^2)**	42<br>(24-61; 86)	62<br>(53-70; 0)	41<br>(26-57; 85)	44<br>(36-52; 61)	39<br>(25-54; 85)	44<br>(24-66; 86)
**Median age 30 or younger**	**N of studies n/N**	4<br>39/108	6<br>124/299	10<br>272/511	6<br>657/1110	2<br>92/131	1<br>36/67
	**Summary % (95% CI; I^2)**	36<br>(22-50; 54)	52<br>(34-70; 88)	53<br>(44-61; 66)	59<br>(55-63; 10)	70<br>(62-78; 82)	54<br>(42-65; NE)
**Median age >30**	**N of studies n/N**	1<br>12/39	NA	2<br>10/51	4<br>134/289	4<br>102/283	1<br>14/25
	**Summary % (95% CI; I^2)**	31<br>(19-46; NE)	NA	19<br>(9-32; 98)	44<br>(32-57; 79)	39<br>(25-54; 85)	56<br>(37-73; NE)
**HR-HPV (+)** [8]	**N of studies n/N**	NA	NA	NA	4<br>87/193	NA	NA
	**Summary % (95% CI; I^2)**	NA	NA	NA	41<br>(26-58; 77)	NA	NA

		Regression 3 months	Regression 6 months	Regression 12 months	Regression 24 months	Regression 36 months	Regression 60 months
**HR-HPV (-)** [8]	**N of studies n/N**	NA	NA	NA	4<br>17/23	NA	NA
	**Summary % (95% CI; I^2)**	NA	NA	NA	62<br>(41-82; 0)	NA	NA
**HPV 16/18 (+)** [8]	**N of studies n/N**	NA	NA	NA	4<br>38/56	NA	NA
	**Summary % (95% CI; I^2)**	NA	NA	NA	42<br>(32-53; 0)	NA	NA
**HPV 16/18 (-)** [8]	**N of studies n/N**	NA	NA	NA	4<br>57/62	NA	NA
	**Summary % (95% CI; I^2)**	NA	NA	NA	71<br>(55-85; 28)	NA	NA

[1] Number of studies included in analysis number of outcomes observed/number of women attended
[2] Summary percentage of outcome (95% confidence interval; I^2 (%))
[3] If more than one definition for regression given be the authors the most stringent definition used (regression defined only as normal histology and/or cytology)
[4] If more than one definition for regression given by the authors the more lenient definition used (regression defined as histological CIN 1 and/or cytological ASC-US or LSIL in addition to normal histology and/or cytology)
[5] Including only studies reporting on exact follow-up time points
[6] Including only studies with lenient definition for regression (regression defined as histological CIN 1 and/or cytological ASC-US or LSIL in addition to normal histology and/or cytology)
[7] Including only studies with strict definition for regression (regression defined only as normal histology and/or cytology)
[8] Baseline HPV status

NA not available in time point

Supplementary table 5. Persistence rates for CIN 2 in all time points and subgroup and sensitivity analyses.

		Persistence 3 months	Persistence 6 months	Persistence 12 months	Persistence 24 months	Persistence 36 months	Persistence 60 months
**Main analysis** [3]	**N of studies n/N** [1]	3<br>56/133	5<br>96/278	9<br>110/414	8<br>334/1257	5<br>102/378	3<br>70/170
	**Summary % (95% CI; I^2)** [2]	47<br>(16-79; 93)	34<br>(29-40; 0)	29<br>(17-43; 85)	32<br>(23-42; 82)	27<br>(20-36; 67)	37<br>(14-64; 91)
**Lenient criteria** [4]	**N of studies n/N**	3<br>38/133	5<br>63/278	9<br>85/414	8<br>334/1257	5<br>102/378	3<br>64/170
	**Summary % (95% CI; I^2)**	28<br>(6-57; 91)	22<br>(17-27; 0)	22<br>(13-34; 82)	32<br>(23-42; 82)	27<br>(20-36; 67)	27<br>(3-62; 95)
**Exact follow-up only** [5]	**N of studies n/N**	2<br>34/100	3<br>29/89	6<br>59/191	2<br>18/107	1<br>16/95	NA
	**Summary % (95% CI; I^2)**	33<br>(24-42; 99)	32<br>(23-43; 0)	31<br>(14-51; 85)	16<br>(9-24; 97)	17<br>(11-26; NE)	NA
**Lenient assesment of outcome** [6]	**N of studies n/N**	2<br>15/94	4<br>50/237	6<br>59/324	6<br>300/1185	NA	1<br>1/25
	**Summary % (95% CI; I^2)**	16<br>(9-24; 98)	21<br>(15-26; 0)	18<br>(12-25; 33)	28<br>(20-38; 79)	NA	4<br>(1-20; NE)
**Strict assessment of outcome** [7]	**N of studies n/N**	2<br>45/72	4<br>91/257	6<br>71/212	2<br>34/72	5<br>102/378	3<br>70/170
	**Summary % (95% CI; I^2)**	63<br>(51-74; 99)	35<br>(29-41; 0)	32<br>(15-52; 88)	47<br>(36-59; 98)	27<br>(20-36; 67)	37<br>(14-64; 91)
**Low risk of bias**	**N of studies n/N**	3<br>56/133	3<br>33/100	5<br>45/149	3<br>275/1049	2<br>29/96	1<br>16/67
	**Summary % (95% CI; I^2)**	47<br>(16-79; 93)	33<br>(24-43; 0)	30<br>(10-56; 89)	35<br>(21-51; 89)	30<br>(21-39; 94)	24<br>(15-35; NE)

		Persistence 3 months	Persistence 6 months	Persistence 12 months	Persistence 24 months	Persistence 36 months	Persistence 60 months
**High risk of bias**	**N of studies n/N**	NA	2<br>63/178	4<br>65/265	5<br>59/208	3<br>73/282	2<br>54/103
	**Summary % (95% CI; I^2)**	NA	35<br>(28-42; 99)	29<br>(14-46; 83)	31<br>(16-48; 81)	26<br>(17-36; 71)	52<br>(43-62; 86)
**Only European studies**	**N of studies n/N**	1<br>11/61	NA	1<br>6/12	1<br>24/51	1<br>22/65	1<br>16/67
	**Summary % (95% CI; I^2)**	18<br>(10-29; NE)	NA	50<br>(25-75; NE)	47<br>(34-60; NE)	34<br>(24-46; NE)	24<br>(15-35; NE)
**Only North America**	**N of studies n/N**	NA	2<br>16/48	5<br>82/302	1<br>10/21	1<br>16/95	1<br>47/78
	**Summary % (95% CI; I^2)**	NA	33<br>(20-47; 99)	31<br>(16-48; 85)	48<br>(28-68; NE)	17<br>(11-26; NE)	60<br>(49-70; NE)
**Only South America**	**N of studies n/N**	2<br>45/72	2<br>22/73	3<br>22/100	1<br>4/14	NA	NA
	**Summary % (95% CI; I^2)**	63<br>(51-74; 98)	30<br>(20-41; 98)	23<br>(1-56; 91)	29<br>(12-55; NE)	NA	NA
**Only Asia**	**N of studies n/N**	NA	NA	NA	4<br>74/247	3<br>64/218	1<br>7/25
	**Summary % (95% CI; I^2)**	NA	NA	NA	30<br>(16-47; 86)	29<br>(20-40; 54)	28<br>(14-48; NE)
**North America excluded**	**N of studies n/N**	3<br>56/133	3<br>80/230	4<br>28/112	7<br>324/1236	4<br>86/283	2<br>23/92
	**Summary % (95% CI; I^2)**	47<br>(16-79; 93)	35<br>(29-41; 0)	28<br>(6-58; 89)	31<br>(22-41; 83)	30<br>(24-38; 38)	25<br>(16-34; 79)
**Europe excluded**	**N of studies n/N**	2<br>45/72	5<br>96/278	8<br>104/402	7<br>310/1206	4<br>80/313	2<br>54/103
	**Summary % (95% CI; I^2)**	63<br>(51-74; 99)	34<br>(29-40; 0)	28<br>(15-42; 86)	30<br>(21-40; 79)	26<br>(17-36; 70)	52<br>(43-62; 90)

		Persistence 3 months	Persistence 6 months	Persistence 12 months	Persistence 24 months	Persistence 36 months	Persistence 60 months
**Only 1980's**	**N of studies n/N**	1 11/61	NA	NA	1 10/21	2 63/145	NA
	**Summary % (95% CI; I^2)**	18 (10-29; NE)	NA	NA	48 (28-68; NE)	43 (35-51; 97)	NA
**Only 1990's**	**N of studies n/N**	NA	NA	2 32/75	2 39/100	1 18/44	NA
	**Summary % (95% CI; I^2)**	NA	NA	42 (31-54; 77)	39 (29-49; 77)	41 (28-56; NE)	NA
**Only 2000's**	**N of studies n/N**	2 45/72	3 80/230	5 45/159	4 264/1082	3 62/269	1 7/25
	**Summary % (95% CI; I^2)**	63 (51-74; 99)	35 (29-41; 0)	27 (8-51; 89)	27 (16-39; 82)	22 (15-30; 53)	28 (14-48; NE)
**Only 2010's**	**N of studies n/N**	NA	2 16/48	2 33/180	1 21/54	NA	NA
	**Summary % (95% CI; I^2)**	NA	33 (20-47; 93)	17 (12-24; 93)	39 (27-52; NE)	NA	NA
**2000's excluded**	**N of studies n/N**	1 11/61	2 16/48	4 65/255	4 70/175	2 40/109	2 63/145
	**Summary % (95% CI; I^2)**	18 (10-29; NE)	33 (20-47; 99)	32 (16-51; 83)	39 (30-49; 32)	37 (28-46; 77)	43 (35-51; 77)
**Only retrospective cohorts**	**N of studies n/N**	NA	2 63/178	4 58/238	6 288/1093	1 35/122	1 47/78
	**Summary % (95% CI; I^2)**	NA	35 (28-42; 99)	31 (13-53; 86)	34 (24-45; 76)	29 (21-37; NE)	60 (49-70; NE)
**Only prospective studies**	**N of studies n/N**	3 56/133	3 33/100	5 52/176	2 46/164	4 67/256	2 23/92
	**Summary % (95% CI; I^2)**	47 (16-79; 93)	33 (24-43; 0)	28 (10-50; 87)	27 (20-34; 97)	27 (17-39; 74)	25 (16-34; 91)

		Persistence 3 months	Persistence 6 months	Persistence 12 months	Persistence 24 months	Persistence 36 months	Persistence 60 months
**Only RCTs**	**N of studies n/N**	1 11/61	1 11/27	1 5/16	NA	NA	NA
	**Summary % (95% CI; I^2)**	18 (10-29; NE)	41 (25-59; NE)	31 (14-56; NE)	NA	NA	NA
**Only prospective cohorts**	**N of studies n/N**	2 45/72	2 22/73	4 47/160	2 46/164	4 67/256	2 23/92
	**Summary % (95% CI; I^2)**	63 (51-74; 99)	30 (20-41; 99)	28 (7-54; 91)	27 (20-34; 97)	27 (17-39; 74)	25 (16-34; 91)
**Upper limit of age range 30 or younger**	**N of studies n/N**	NA	3 74/205	5 63/254	2 226/938	1 16/95	NA
	**Summary % (95% CI; I^2)**	NA	36 (29-43; 0)	31 (15-49; 82)	23 (20-26; 97)	17 (11-26; NE)	NA
**Upper limit of age range >30**	**N of studies n/N**	3 56/133	2 22/73	4 47/160	6 108/319	4 86/283	3 70/170
	**Summary % (95% CI; I^2)**	47 (16-79; 93)	30 (20-41; 97)	28 (7-54; 91)	35 (23-49; 83)	30 (24-38; 38)	37 (14-64; 91)
**Median age 30 or younger**	**N of studies n/N**	2 45/72	5 96/278	8 104/402	3 236/959	1 16/95	1 16/67
	**Summary % (95% CI; I^2)**	63 (51-74; 99)	34 (29-40; 0)	28 (15-42; 86)	31 (17-47; 63)	17 (11-26; NE)	24 (15-35; NE)
**Median age >30**	**N of studies n/N**	NA	NA	1 6/12	3 59/198	4 86/283	1 7/25
	**Summary % (95% CI; I^2)**	NA	NA	50 (25-75; NE)	32 (13-55; 90)	30 (24-38; 38)	28 (14-48; NE)
**HR-HPV (+) [8]**	**N of studies n/N**	NA	NA	NA	3 36/139	NA	NA
	**Summary % (95% CI; I^2)**	NA	NA	NA	33 (11-59; 86)	NA	NA

		Persistence 3 months	Persistence 6 months	Persistence 12 months	Persistence 24 months	Persistence 36 months	Persistence 60 months
**HR-HPV (-)** [8]	**N of studies n/N**	NA	NA	NA	3<br>5/23	NA	NA
	**Summary % (95% CI; I^2)**	NA	NA	NA	32<br>(7-61; 0)	NA	NA
**HPV 16/18 (+)** [8]	**N of studies n/N**	NA	NA	NA	2<br>11/56	NA	NA
	**Summary % (95% CI; I^2)**	NA	NA	NA	32<br>(17-50; 58)	NA	NA
**HPV 16/18 (-)** [8]	**N of studies n/N**	NA	NA	NA	2<br>4/62	NA	NA
	**Summary % (95% CI; I^2)**	NA	NA	NA	30<br>(7-60; 76)	NA	NA

[1] Number of studies included in analysis number of outcomes observed/number of women attended
[2] Summary percentage of outcome (95% confidence interval; I^2 (%))
[3] If more than one definition for persistence given be the authors the most stringent definition used (persistence defined as histological CIN 2 or CIN 1 and/or cytological HSIL or ASC-H or LSIL or ASC-US)
[4] If more than one definition for persistence given by the authors the more lenient definition used (persistence defined as histological CIN 2 and/or cytological HSIL or ASC-H)
[5] Including only studies reporting on exact follow-up time points
[6] Including only studies with lenient definition for persistence (persistence defined as histological CIN 2 and/or cytological HSIL or ASC-H)
[7] Including only studies with strict definition for persistence (persistence defined as histological CIN 2 or CIN 1 and/or cytological HSIL or ASC-H or LSIL or ASC-US)
[8] Baseline HPV status

NA not available in time point

Supplementary table 6. Progression rates for CIN 2 in all time points and subgroup and sensitivity analyses.

		Progression 3 months	Progression 6 months	Progression 12 months	Progression 24 months	Progression 36 months	Progression 60 months
**Main analysis**	**N of studies n/N [1]**	3 7/133	5 42/278	13 131/834	9 282/1445	3 105/370	6 172/544
	**Summary % (95% CI; I^2) [2]**	5 (2-10; 0)	13 (8-20; 42)	14 (9-20; 75)	18 (11-27; 90)	24 (12-39; 87)	23 (10-39; 93)
**Lenient criteria**	**N of studies n/N**	NA	NA	NA	NA	NA	NA
	**Summary % (95% CI; I^2)**	NA	NA	NA	NA	NA	NA
**Exact follow-up only [3]**	**N of studies n/N**	2 5/100	3 11/89	7 30/286	3 35/202	1 14/95	NA
	**Summary % (95% CI; I^2)**	5 (1-10; 98)	13 (3-26; 58)	10 (3-21; 80)	12 (2-27; 83)	15 (9-23; NE)	NA
**Lenient assesment of outcome**	**N of studies n/N**	NA	NA	NA	NA	NA	NA
	**Summary % (95% CI; I^2)**	NA	NA	NA	NA	NA	NA
**Strict assessment of outcome**	**N of studies n/N**	NA	NA	NA	NA	NA	NA
	**Summary % (95% CI; I^2)**	NA	NA	NA	NA	NA	NA
**Low risk of bias**	**N of studies n/N**	3 7/133	3 9/100	6 66/380	3 181/1049	2 91/275	4 157/441
	**Summary % (95% CI; I^2)**	5 (2-10; 0)	9 (4-15; 0)	17 (12-21; 5)	20 (12-30; 76)	33 (27-39; 88)	27 (11-47; 94)

		Progression 3 months	Progression 6 months	Progression 12 months	Progression 24 months	Progression 36 months	Progression 60 months
**High risk of bias**	**N of studies n/N**	NA	2<br>33/178	7<br>65/454	6<br>101/396	1<br>14/95	2<br>15/103
	**Summary % (95% CI; I^2)**	NA	18<br>(12-24; 99)	11<br>(4-21; 85)	16<br>(5-32; 92)	15<br>(9-23; NE)	14<br>(8-22; 98)
**Only European studies**	**N of studies n/N**	1<br>4/61	NA	2<br>16/77	1<br>5/51	NA	1<br>13/67
	**Summary % (95% CI; I^2)**	7<br>(3-16; NE)	NA	20<br>(11-30; 88)	10<br>(4-21; NE)	NA	19<br>(12-30; NE)
**Only North America**	**N of studies n/N**	NA	2<br>9/48	6<br>50/397	2<br>13/116	1<br>14/95	1<br>11/78
	**Summary % (95% CI; I^2)**	NA	19<br>(8-31; 97)	10<br>(3-20; 84)	11<br>(5-17; 90)	15<br>(9-23; NE)	14<br>(8-24; NE)
**Only South America**	**N of studies n/N**	2<br>3/72	2<br>5/73	3<br>20/100	1<br>0/14	NA	NA
	**Summary % (95% CI; I^2)**	4<br>(0-10; 95)	7<br>(2-14; 95)	15<br>(3-32; 75)	0<br>(0-22; NE)	NA	NA
**Only Asia**	**N of studies n/N**	NA	NA	2<br>45/260	4<br>112/340	2<br>91/275	4<br>148/399
	**Summary % (95% CI; I^2)**	NA	NA	17<br>(12-22; 99)	30<br>(17-46; 88)	33<br>(27-39; 96)	26<br>(9-48; 94)
**North America excluded**	**N of studies n/N**	3<br>7/133	3<br>33/230	7<br>81/437	7<br>269/1329	2<br>91/275	5<br>161/466
	**Summary % (95% CI; I^2)**	5<br>(2-10; 0)	11<br>(4-21; 63)	18<br>(13-24; 39)	21<br>(11-32; 92)	33<br>(27-39; 99)	25<br>(11-43; 93
**Europe excluded**	**N of studies n/N**	2<br>3/72	5<br>42/278	11<br>115/757	8<br>277/1394	3<br>105/370	5<br>159/477
	**Summary % (95% CI; I^2)**	4<br>(0-10; 100)	13<br>(8-20; 42)	13<br>(7-19; 78)	20<br>(11-30; 90)	24<br>(12-39; 87)	24<br>(9-42; 94)

		Progression 3 months	Progression 6 months	Progression 12 months	Progression 24 months	Progression 36 months	Progression 60 months
**Only 1980's**	**N of studies n/N**	1 4/61	NA	NA	1 2/21	NA	2 24/145
	**Summary % (95% CI; I^2)**	7 (3-16; NE)	NA	NA	10 (3-29; NE)	NA	16 (11-23; 79)
**Only 1990's**	**N of studies n/N**	NA	NA	3 13/104	1 11/71	1 10/44	NA
	**Summary % (95% CI; I^2)**	NA	NA	15 (2-35; 77)	15 (9-26; NE)	23 (13-37; NE)	NA
**Only 2000's**	**N of studies n/N**	2 3/72	3 33/230	7 67/485	6 251/1299	2 95/326	4 148/399
	**Summary % (95% CI; I^2)**	4 (0-10; 100)	11 (4-21; 63)	12 (5-20; 80)	18 (8-30; 93)	29 (24-34; 99)	26 (9-48; 94)
**Only 2010's**	**N of studies n/N**	NA	2 9/48	2 38/180	1 18/54	NA	NA
	**Summary % (95% CI; I^2)**	NA	19 (8-31; 79)	20 (15-27; 79)	33 (22-47; NE)	NA	NA
**2000's excluded**	**N of studies n/N**	1 4/61	2 9/48	6 64/349	3 31/146	1 10/44	2 24/145
	**Summary % (95% CI; I^2)**	7 (3-16; NE)	19 (8-31; 98)	17 (9-25; 64)	20 (8-34; 72)	23 (13-37; NE)	16 (11-23; 86)
**Only retrospective cohorts**	**N of studies n/N**	NA	2 33/178	5 50/267	6 236/1186	NA	1 11/78
	**Summary % (95% CI; I^2)**	NA	18 (12-24; 99)	14 (6-24; 63)	18 (7-33; 93)	NA	14 (8-24; NE)
**Only prospective studies**	**N of studies n/N**	3 7/133	3 9/100	8 81/567	3 46/259	3 105/370	5 161/466
	**Summary % (95% CI; I^2)**	5 (2-10; 0)	9 (4-15; 0)	14 (7-22; 80)	17 (10-27; 69)	24 (12-39; 87)	25 (11-43; 93)

		Progression 3 months	Progression 6 months	Progression 12 months	Progression 24 months	Progression 36 months	Progression 60 months
**Only RCTs**	**N of studies n/N**	1<br>4/61	1<br>4/27	1<br>2/16	NA	NA	NA
	**Summary % (95% CI; I^2)**	7<br>(3-16; NE)	15<br>(6-32; NE)	12<br>(3-36; NE)	NA	NA	NA
**Only prospective cohorts**	**N of studies n/N**	2<br>3/72	2<br>5/73	7<br>79/551	3<br>46/259	3<br>105/370	5<br>161/466
	**Summary % (95% CI; I^2)**	4<br>(0-10; 100)	7<br>(2-14; 100)	14<br>(7-23; 83)	17<br>(10-27; 69)	24<br>(12-39; 87)	25<br>(11-43; 93)
**Upper limit of age range 30 or younger**	**N of studies n/N**		3<br>37/205	6<br>47/349	3<br>163/1033	1<br>14/95	
	**Summary % (95% CI; I^2)**		18<br>(12-23; 0)	9<br>(2-20; 84)	11<br>(5-19; 67)	15<br>(9-23; NE)	
**Upper limit of age range >30**	**N of studies n/N**	3<br>7/133	2<br>5/73	7<br>84/485	6<br>119/412	2<br>91/275	6<br>172/544
	**Summary % (95% CI; I^2)**	5<br>(2-10; 0)	7<br>(2-14; 0)	17<br>(12-24; 58)	23<br>(12-37; 89)	33<br>(27-39; 98)	23<br>(10-39; 93)
**Median age 30 or younger**	**N of studies n/N**	2<br>3/72	5<br>42/278	9<br>70/497	4<br>165/1054	1<br>14/95	1<br>13/67
	**Summary % (95% CI; I^2)**	4<br>(0-10; 99)	13<br>(8-20; 42)	11<br>(5-19; 81)	11<br>(6-18; 53)	15<br>(9-23; NE)	19<br>(12-30; NE)
**Median age >30**	**N of studies n/N**	NA	NA	3<br>54/308	4<br>106/320	2<br>91/275	4<br>148/399
	**Summary % (95% CI; I^2)**	NA	NA	17<br>(13-21; 0)	29<br>(14-46; 90)	33<br>(27-39; 97)	26<br>(9-48; 94)
**HR-HPV (+)** [4]	**N of studies n/N**	NA	NA	NA	3<br>38/139	NA	NA
	**Summary % (95% CI; I^2)**	NA	NA	NA	25<br>(14-38; 51)	NA	NA

		Progression 3 months	Progression 6 months	Progression 12 months	Progression 24 months	Progression 36 months	Progression 60 months
**HR-HPV (-) [4]**	**N of studies n/N**	NA	NA	NA	3<br>1/23	NA	NA
	**Summary % (95% CI; I^2)**	NA	NA	NA	3<br>(0-24; 0)	NA	NA
**HPV 16/18 (+) [4]**	**N of studies n/N**	NA	NA	NA	2<br>7/56	NA	NA
	**Summary % (95% CI; I^2)**	NA	NA	NA	21<br>(8-37; 58)	NA	NA
**HPV 16/18 (-) [4]**	**N of studies n/N**	NA	NA	NA	2<br>1/62	NA	NA
	**Summary % (95% CI; I^2)**	NA	NA	NA	5<br>(0-28; 76)	NA	NA

[1] Number of studies included in analysis number of outcomes observed/number of women attended
[2] Summary percentage of outcome (95% confidence interval; I^2 (%))
[3] Including only studies reporting on exact follow-up time points
[4] Baseline HPV status

NA not available in time point

Supplementary Table 7. Default rates for CIN 2 in all time points and subgroup and sensitivity analyses.

		Default 3 months	Default 6 months	Default 12 months	Default 24 months	Default 36 months
**Main analysis**	**N of studies n/N** [1]	4 12/138	5 69/316	9 120/564	6 61/439	3 25/216
	**Summary % (95% CI; I^2)** [2]	4 (0-17; 83)	19 (7-35; 88)	15 (6-25; 87)	8 (1-21; 92)	4 (0-23; 94)
**Lenient criteria**	**N of studies n/N**	NA	NA	NA	NA	NA
	**Summary % (95% CI; I^2)**	NA	NA	NA	NA	NA
**Exact follow-up only** [3]	**N of studies n/N**	4 12/138	5 69/316	7 73/339	4 57/313	3 25/216
	**Summary % (95% CI; I^2)**	4 (0-17; 83)	19 (7-35; 88)	16 (5-30; 89)	13 (2-29; 91)	4 (0-23; 94)
**Lenient assesment of outcome**	**N of studies n/N**	NA	NA	NA	NA	NA
	**Summary % (95% CI; I^2)**	NA	NA	NA	NA	NA
**Strict assessment of outcome**	**N of studies n/N**	NA	NA	NA	NA	NA
	**Summary % (95% CI; I^2)**	NA	NA	NA	NA	NA
**Low risk of bias**	**N of studies n/N**	4 12/138	3 13/113	5 14/161	2 0/125	2 0/96
	**Summary % (95% CI; I^2)**	4 (0-17; 83)	10 (4-19; 37)	6 (1-13; 42)	0 (0-2; 86)	0 (0-2; 86)

		Default 3 months	Default 6 months	Default 12 months	Default 24 months	Default 36 months
**High risk of bias**	**N of studies n/N**	NA	2<br>56/203	4<br>106/403	4<br>61/314	1<br>25/120
	**Summary % (95% CI; I^2)**	NA	27<br>(21-33; 98)	28<br>(14-45; 90)	17<br>(10-26; 63)	21<br>(15-29; NE)
**Only European studies**	**N of studies n/N**	1<br>0/39	NA	2<br>0/26	1<br>4/55	NA
	**Summary % (95% CI; I^2)**	0<br>(0-9; NE)	NA	0<br>(0-7; 77)	7<br>(3-17; NE)	NA
**Only North America**	**N of studies n/N**	1<br>0/15	2<br>27/75	4<br>108/426	1<br>25/120	1<br>25/120
	**Summary % (95% CI; I^2)**	0<br>(0-20; NE)	33<br>(23-45; 99)	26<br>(13-43; 91)	21<br>(15-29; NE)	21<br>(15-29; NE)
**Only South America**	**N of studies n/N**	2<br>12/84	2<br>11/84	3<br>12/112	1<br>3/17	NA
	**Summary % (95% CI; I^2)**	13<br>(6-21; 93)	12<br>(6-21; 93)	10<br>(3-19; 42)	18<br>(6-41; NE)	NA
**Only Asia**	**N of studies n/N**	NA	NA	NA	3<br>29/247	2<br>0/96
	**Summary % (95% CI; I^2)**	NA	NA	NA	4<br>(0-26; 96)	0<br>(0-2; 98)
**North America excluded**	**N of studies n/N**	3<br>12/123	3<br>42/241	5<br>12/138	5<br>36/319	2<br>0/96
	**Summary % (95% CI; I^2)**	6<br>(0-24; 88)	15<br>(8-24; 53)	6<br>(1-14; 44)	6<br>(0-21; 92)	0<br>(0-2; 98)
**Europe excluded**	**N of studies n/N**	3<br>12/99	5<br>69/316	7<br>120/538	5<br>57/384	3<br>25/216
	**Summary % (95% CI; I^2)**	7<br>(0-24; 80)	19<br>(7-35; 88)	19<br>(10-31; 88)	9<br>(0-24; 94)	4<br>(0-23; 94)

		Default 3 months	Default 6 months	Default 12 months	Default 24 months	Default 36 months
**Only 1980's**	**N of studies n/N**	NA	NA	1<br>0/14	NA	NA
	**Summary % (95% CI; I^2)**	NA	NA	0<br>(0-22; NE)	NA	NA
**Only 1990's**	**N of studies n/N**	1<br>0/15	NA	1<br>0/12	1<br>0/71	1<br>0/44
	**Summary % (95% CI; I^2)**	0<br>(0-20; NE)	NA	0<br>(0-24; NE)	0<br>(0-5; NE)	0<br>(0-8; NE)
**Only 2000's**	**N of studies n/N**	3<br>12/123	3<br>42/241	6<br>73/327	4<br>61/314	2<br>25/172
	**Summary % (95% CI; I^2)**	6<br>(0-24; 88)	15<br>(8-24; 53)	19<br>(7-35; 90)	17<br>(10-26; 63)	11<br>(7-17; 88)
**Only 2010's**	**N of studies n/N**	NA	2<br>27/75	1<br>47/211	1<br>0/54	NA
	**Summary % (95% CI; I^2)**	NA	33<br>(23-45; 98)	22<br>(17-28; NE)	0<br>(0-7; NE)	NA
**2000's excluded**	**N of studies n/N**	1<br>0/15	2<br>27/75	3<br>47/237	2<br>0/125	1<br>0/44
	**Summary % (95% CI; I^2)**	0<br>(0-20; NE)	33<br>(23-45; 99)	5<br>(0-27; 84)	0<br>(0-2; 95)	0<br>(0-8; NE)
**Only retrospective cohorts**	**N of studies n/N**	NA	2<br>56/203	4<br>85/323	3<br>7/126	NA
	**Summary % (95% CI; I^2)**	NA	27<br>(21-33; 99)	25<br>(8-46; 91)	5<br>(0-19; 80)	NA
**Only prospective studies**	**N of studies n/N**	4<br>12/138	3<br>13/113	5<br>35/241	3<br>54/313	3<br>25/216
	**Summary % (95% CI; I^2)**	4<br>(0-17; 83)	10<br>(4-19; 37)	8<br>(1-18; 74)	11<br>(0-33; 95)	4<br>(0-23; 94)

		Default 3 months	Default 6 months	Default 12 months	Default 24 months	Default 36 months
**Only RCTs**	**N of studies n/N**	1<br>0/15	1<br>2/29	NA	NA	NA
	**Summary % (95% CI; I^2)**	0<br>(0-20; NE)	7<br>(2-22; NE)	NA	NA	NA
**Only prospective cohorts**	**N of studies n/N**	3<br>12/123	2<br>11/84	5<br>35/241	3<br>54/313	3<br>25/216
	**Summary % (95% CI; I^2)**	6<br>(0-24; 88)	12<br>(6-21; 94)	8<br>(1-18; 74)	11<br>(0-33; 95)	4<br>(0-23; 94)
**Upper limit of age range 30 or younger**	**N of studies n/N**	NA	3<br>58/232	5<br>110/443	2<br>28/137	1<br>25/120
	**Summary % (95% CI; I^2)**	NA	25<br>(6-52; 92)	24<br>(12-38; 89)	20<br>(13-27; 97)	21<br>(15-29; NE)
**Upper limit of age range >30**	**N of studies n/N**	4<br>12/138	2<br>11/84	4<br>10/121	4<br>33/302	2<br>0/96
	**Summary % (95% CI; I^2)**	4<br>(0-17; 83)	12<br>(6-21; 94)	5<br>(0-14; 54)	5<br>(0-20; 94)	0<br>(0-2; 98)
**Median age 30 or younger**	**N of studies n/N**	3<br>12/99	5<br>69/316	8<br>120/552	2<br>28/137	1<br>25/120
	**Summary % (95% CI; I^2)**	7<br>(0-24; 80)	19<br>(7-35; 88)	17<br>(8-28; 88)	20<br>(13-27; 98)	21<br>(15-29; NE)
**Median age >30**	**N of studies n/N**	1<br>0/39	NA	1<br>0/12	3<br>33/231	2<br>0/96
	**Summary % (95% CI; I^2)**	0<br>(0-9; NE)	NA	0<br>(0-24; NE)	8<br>(0-28; 94)	0<br>(0-2; 97)

[1] Number of studies included in analysis number of outcomes observed/number of women attended
[2] Summary percentage of outcome (95% confidence interval; I^2 (%))
[3] Including only studies reporting on exact follow-up time points

NA not available in time point
